# Cannabidiol-induced crosstalk of apoptosis and macroautophagy in colorectal cancer cells involves p53 and Hsp70

**DOI:** 10.1038/s41420-023-01578-9

**Published:** 2023-08-05

**Authors:** Fei Wang, Ali Bashiri Dezfouli, Mohammad Khosravi, Wolfgang Sievert, Stefan Stangl, Melissa Schwab, Zhiyuan Wu, Katja Steiger, Hu Ma, Gabriele Multhoff

**Affiliations:** 1 Radiation Immuno-Oncology Project Group, TranslaTUM—Central Institute for Translational Cancer Research, Klinikum rechts der Isar, Technische Universität München, Munich, Germany; 2 Department of Oncology, The Second Affiliated Hospital of Zunyi Medical University, Zunyi, China; 3 Department of Pathobiology, Faculty of Veterinary Medicine, Shahid Chamran University of Ahvaz, Ahvaz, Iran; 4 Institute for General Pathology and Pathological Anatomy, Technische Universität München, Munich, Germany

**Keywords:** Chemotherapy, Drug regulation

## Abstract

Although it has been established that cannabidiol (CBD), the major non-psychoactive constituent of cannabis, exerts antitumoral activities, the exact mechanism(s) via which tumor cells are killed by CBD are not well understood. This study provides new insights into the potential mechanisms of CBD-induced mutual antagonism of apoptosis and macroautophagy using wild type (HCT116 p53wt, LS174T p53wt), knockout (HCT116 p53^−/−^) and mutant (SW480 p53mut) human colorectal cancer cells (CRC). CBD causes a more pronounced loss in the viability of p53wt cells than p53^−/−^ and p53mut cells, and a 5-week treatment with CBD reduced the volume of HCT116 p53wt xenografts in mice, but had no effect on the volume of HCT116 p53^−/−^ tumors. Mechanistically, we demonstrate that CBD only significantly elevates ROS production in cells harboring wild-type p53 (HCT116, LS174T) and that this is associated with an accumulation of PARP1. CBD-induced elevated ROS levels trigger G0/G1 cell cycle arrest, a reduction in CDK2, a p53-dependent caspase-8/9/3 activation and macroautophagy in p53wt cells. The ROS-induced macroautophagy which promotes the activation of keap1/Nrf2 pathway might be positively regulated by p53wt, since inhibition of p53 by pifithrin-α further attenuates autophagy after CBD treatment. Interestingly, an inhibition of heat shock protein 70 (Hsp70) expression significantly enhances caspase-3 mediated programmed cell death in p53wt cells, whereas autophagy—which is associated with a nuclear translocation of Nrf2—was blocked. Taken together, our results demonstrate an intricate interplay between apoptosis and macroautophagy in CBD-treated colorectal cancer cells, which is regulated by the complex interactions of p53wt and Hsp70.

## Introduction

Colorectal cancer (CRC) is one of the most common malignant tumors in the gastrointestinal tract and the third leading cause of cancer-related deaths worldwide. Adenocarcinoma is the most common pathohistological type and accounts for more than 90% of all CRC cases [1]. Although advanced chemotherapeutic concepts such as FOLFOX or FOLFIRI in combination with molecular targeted drugs have achieved better clinical outcomes, the 5-year survival rate remains at only 12.5% [2], and acquired resistance to therapy occurs in 90% of CRC patients with metastatic disease.

Genomic instability, including microsatellite instability (MSI), chromosomal instability (CIN), and chromosome translocations play crucial roles in the etiology of CRC [3]. Mutations in tumor suppressor genes such as adenomatous polyposis coli (APC) and TP53, as well as an inactive epigenetic mismatch repair (MMR) system and a genetic mutation in MutL Homolog 1 (MLH1) [3, 4] are also involved in tumorigenesis. The TP53 gene encodes for the p53 tumor suppressor protein that regulates a large variety of different cellular processes such as apoptosis, senescence, cell-cycle arrest and metabolism [5]. TP53 is the most frequently mutated gene in multiple human malignancies, including colon adenocarcinoma, and 60% of all colon cancers are associated with mutations in the TP53 gene locus [6, 7] that frequently result in a functional loss of p53 in later stages of cancer progression [8].

Cannabidiol (CBD), one of the phytocannabinoids of *Cannabis sativa L*., is well-tolerated and has antitumoral properties [9]. CBD inhibits invasion and metastasis of non-small cell lung cancer (NSCLC) cells by lowering plasminogen activator inhibitor-1 (PAI-1) levels [10]. CBD is a selective activator of TRPV2 [11] and induces apoptosis in U87 glioblastoma cells by enhancing the Ca^2+^ influx [12]. Furthermore, attenuating the activity of EGF/EGFR signaling pathway and its client kinase Akt, ERK by CBD in multiple tumor cell types [13, 14] triggers programmed cell death [13] and inhibits angiogenesis and invasive growth [14].

Although multiple antitumoral activities have been described for CBD, the interaction of p53 with CBD and its consequences on tumor cell death remain to be elucidated. In this study, we have shown that the antitumoral activity of CBD is dependent on p53wt in different colon carcinoma cells and that Hsp70 plays a key role in the decision of colon cancer cells to undergo apoptosis or autophagy. A better understanding of the anti-tumorigenic mechanisms and pathways induced by CBD will assist the design of more effective, combined therapeutic strategies for CRC.

## Results

### Screening for potential targets and pathways induced by CBD in CRC

**Fig. 1:**
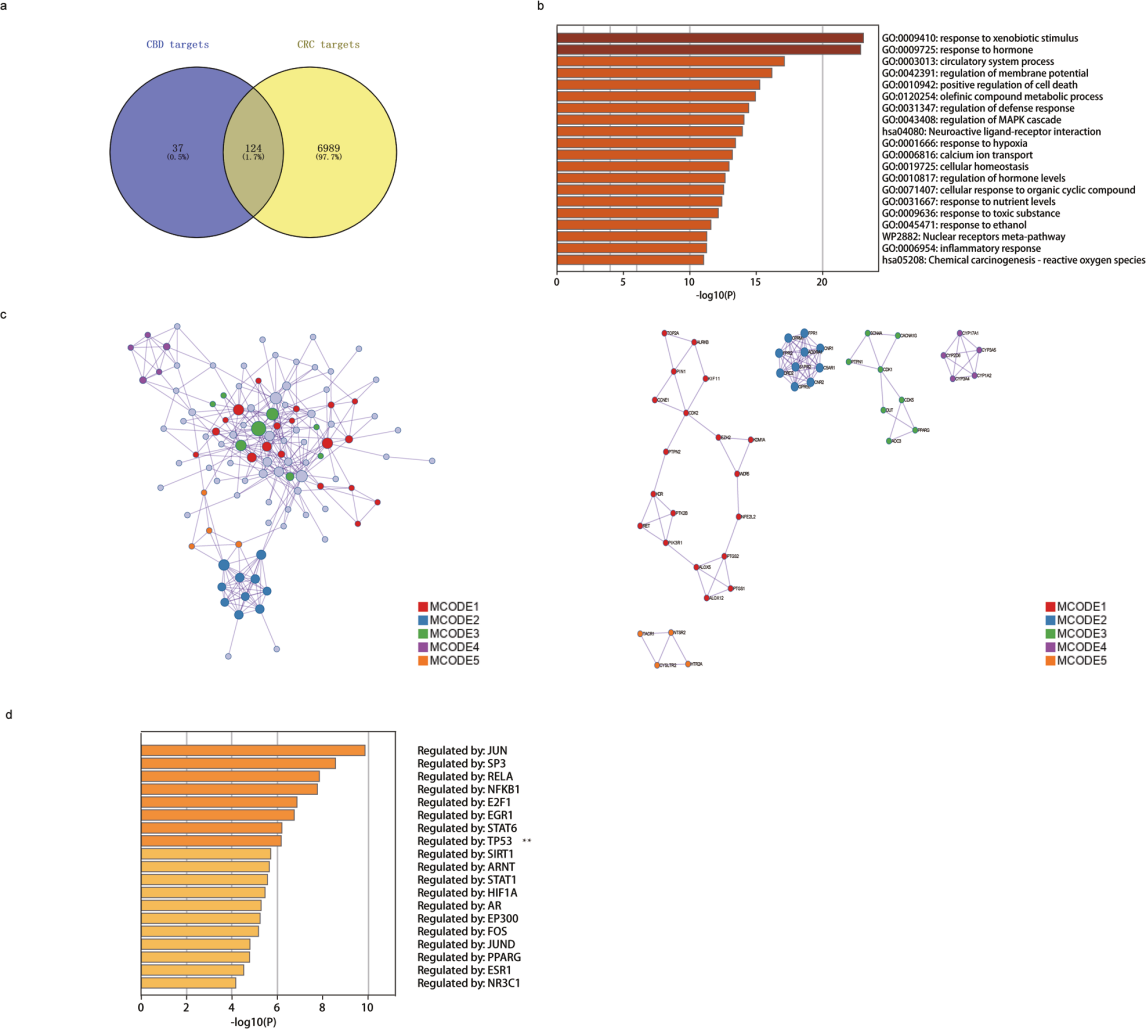
Screening of potential targets of CBD based on the target gene enrichment assay. **a** The Venn diagram identifies overlapping target genes affected in CRC after CBD treatment. **b** Hierarchy of 124 overlapping target genes acquired from the Venn analysis based on the *p* values of the Pathway and Process Enrichment Analysis. **c** Protein–protein interaction network and identified MCODE components. **d** List of most affected genes identified by the TRRUST database.

By importing the 2D molecular structure files of CBD (SMILES:CCCCCC1=CC(=C(C(=C1)O)C2C=C(CCC2C(=C)C)C)O) from the PubChem Database (http://pubchem.ncbi.nlm.nih.gov/) [15] into the SwissTargetPrediction (probability score >0.9 as potential targets), Drugbank and SuperPred databases a total of 161 gene products that potentially interact with CBD were identified. The GeneCard (https://www.genecards.org/) database was used to identify CRC-related targets which are associated with the treatment of CRC using a relevance score ≥10. The Veeny 2.1 (https://bioinfogp.cnb.csic.es/tools/venny/) intersection program (Fig. 1a) identified 124 overlapping target genes that play a role in CRC after treatment with CBD. Metascape were used for pathway and process enrichment analysis (Fig. 1b and Table 1), Protein–Protein Interaction (PPI) network map (Fig. 1c and Table 2) and Predicted Transcriptional Process Enrichment analysis (Fig. 1d and Table 3) of potential target genes involved in the interaction of CBD with CRC. Pathway and process enrichment analysis were performed with the following ontology sources: KEGG Pathway, GO Biological Processes, Reactome Gene Sets, Canonical Pathways, CORUM, WikiPathways and PANTHER Pathway. Transcriptional process enrichment analysis was undertaken using the TRRUST database [16]. Only terms with a *p* value < 0.01, a minimum count of 3, and an enrichment factor >1.5 (the enrichment factor is the ratio between the observed counts and the counts expected by chance) were considered and grouped into clusters based on their membership similarities. Based on target gene enrichment assay, the TP53 regulation was identified as one of the most commonly affected transcriptional processes after treatment of CRC with CBD (Log10(*p*) = −6.5) (Fig. 1d and Table 3).

**Table 1:** Pathway and process enrichment analysis.

GO	Category	Description	Count	%	Log10(*p*)	Log10(*q*)
GO:0009410	GO Biological Processes	Response to xenobiotic stimulus	26	20.97	−23.07	−18.81
GO:0009725	GO Biological Processes	Response to hormone	32	25.81	−22.86	−18.81
GO:0003013	GO Biological Processes	Circulatory system process	23	18.55	−17.12	−13.55
GO:0042391	GO Biological Processes	Regulation of membrane potential	21	16.94	−16.18	−12.79
GO:0010942	GO Biological Processes	Positive regulation of cell death	23	18.55	−15.27	−11.98
GO:0120254	GO Biological Processes	Olefinic compound metabolic process	14	11.29	−14.94	−11.74
GO:0031347	GO Biological Processes	Regulation of defense response	23	18.55	−14.43	−11.29
GO:0043408	GO Biological Processes	Regulation of MAPK cascade	23	18.55	−14.08	−10.98
hsa04080	KEGG Pathway	Neuroactive ligand-receptor interaction	18	14.52	−13.95	−10.89
GO:0001666	GO Biological Processes	Response to hypoxia	16	12.9	−13.44	−10.47
GO:0006816	GO Biological Processes	Calcium ion transport	15	12.1	−13.2	−10.28
GO:0019725	GO Biological Processes	Cellular homeostasis	21	16.94	−12.95	−10.1
GO:0010817	GO Biological Processes	Regulation of hormone levels	19	15.32	−12.65	−9.82
GO:0071407	GO Biological Processes	Cellular response to organic cyclic compound	19	15.32	−12.55	−9.76
GO:0031667	GO Biological Processes	Response to nutrient levels	18	14.52	−12.41	−9.65
GO:0009636	GO Biological Processes	Response to toxic substance	14	11.29	−12.14	−9.42
GO:0045471	GO Biological Processes	Response to ethanol	11	8.87	−11.59	−8.97
WP2882	WikiPathways	Nuclear receptors meta-pathway	15	12.10	−11.28	−8.70
GO:0006954	GO Biological Processes	Inflammatory response	18	14.52	−11.26	−8.69
hsa05208	KEGG Pathway	Chemical carcinogenesis—reactive oxygen species	13	10.48	−11.04	−8.48

Top 20 clusters with their representative enriched terms (one per cluster). “Count” is the number of genes in the user-provided lists with membership in the given ontology term. “%” is the percentage of all of the user-provided genes that are found in the given ontology term (only input genes with at least one ontology term annotation are included in the calculation). “Log10(*p*)” is the *p* value in log base 10. “Log10(*q*)” is the multi-test adjusted *p* value in log base 10.

**Table 2:** Protein–protein interaction enrichment analysis.

MCODE	GO	Description	Log10(*p*)
MCODE_1	R-HSA-9018683	Biosynthesis of DPA-derived SPMs	−9.1
MCODE_1	R-HSA-9025094	Biosynthesis of DPAn-3 SPMs	−9.1
MCODE_1	WP167	Eicosanoid synthesis	−8.7
MCODE_2	R-HSA-373076	Class A/1 (Rhodopsin-like receptors)	−19.6
MCODE_2	R-HSA-500792	GPCR ligand binding	−18.1
MCODE_2	R-HSA-418594	G alpha (i) signaling events	−16.9
MCODE_3	GO:0051223	Regulation of protein transport	−5.3
MCODE_3	GO:0070201	Regulation of establishment of protein localization	−5.2
MCODE_3	GO:0033157	Regulation of intracellular protein transport	−4.7
MCODE_4	GO:0009820	Alkaloid metabolic process	−14
MCODE_4	WP43	Oxidation by cytochrome P450	−13.5
MCODE_4	GO:0070989	Oxidative demethylation	−12.3
MCODE_5	R-HSA-416476	G alpha (q) signaling events	−8.6
MCODE_5	R-HSA-373076	Class A/1 (Rhodopsin-like receptors)	−7.8
MCODE_5	hsa04080	Neuroactive ligand-receptor interaction	−7.7

Protein–protein interaction enrichment analysis has been carried out with the STRING [66] (physical score > 0.132), and BioGrid [67] were used. The Molecular Complex Detection (MCODE) algorithm [68] has been applied to identify densely connected network components. “Log10(*p*)” is the *p* value in Log base 10.

**Table 3:** Summary of enrichment analysis in TRRUST.

GO	Description	Count	%	Log10(*p*)	Log10(*q*)
TRR00645	Regulated by: JUN	11	8.9	−9.9	−7.8
TRR01259	Regulated by: SP3	9	7.3	−8.6	−6.6
TRR01158	Regulated by: RELA	12	9.7	−7.9	−6
TRR00875	Regulated by: NFKB1	12	9.7	−7.8	−5.9
TRR00230	Regulated by: E2F1	8	6.5	−6.9	−5.1
TRR00253	Regulated by: EGR1	7	5.6	−6.7	−5
TRR01282	Regulated by: STAT6	5	4	−6.2	−4.5
TRR01419	Regulated by: TP53	8	6.5	−6.2	−4.5
TRR01225	Regulated by: SIRT1	5	4	−5.7	−4.1
TRR00015	Regulated by: ARNT	4	3.2	−5.6	−4
TRR01275	Regulated by: STAT1	6	4.8	−5.6	−4
TRR00484	Regulated by: HIF1A	6	4.8	−5.5	−3.9
TRR00011	Regulated by: AR	6	4.8	−5.3	−3.7
TRR00270	Regulated by: EP300	5	4	−5.2	−3.7
TRR00342	Regulated by: FOS	5	4	−5.2	−3.6
TRR00647	Regulated by: JUND	4	3.2	−4.8	−3.3
TRR01062	Regulated by: PPARG	5	4	−4.8	−3.3
TRR00275	Regulated by: ESR1	5	4	−4.5	−3
TRR00908	Regulated by: NR3C1	4	3.2	−4.2	−2.7

“Count” is the number of genes with membership in the given ontology term. “%” is the percentage of genes that are found in the given ontology term (only input genes with at least one ontology term annotation are included in the calculation). “Log10(*p*)” is the *p* value in log base 10. “Log10(*q*)” is the multi-test adjusted *p* value in Log base 10.

### p53-dependent reduction in viability and growth of CRC cells in vitro and in vivo

**Fig. 2:**
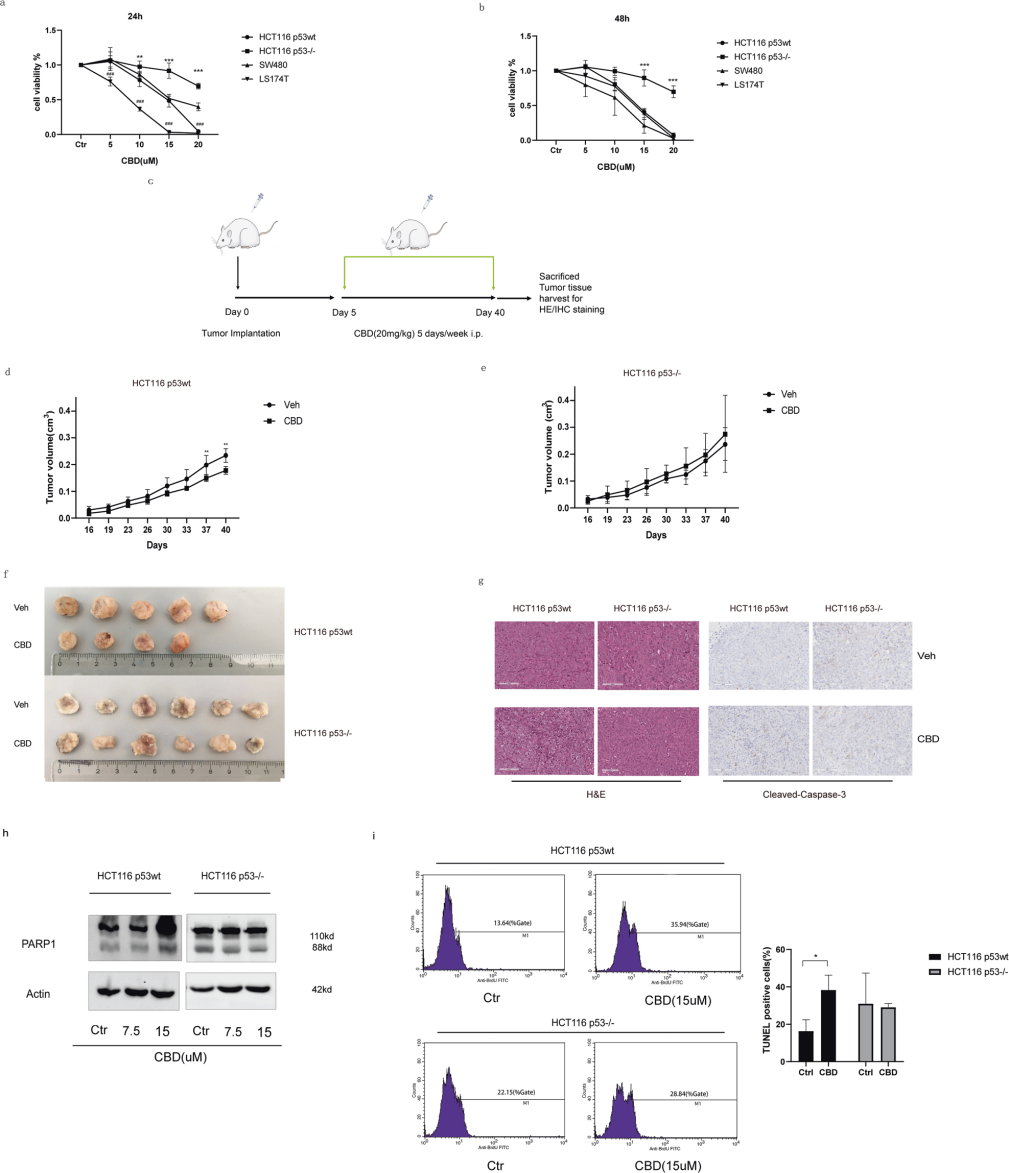
CBD reduces the viability of p53wt CRC cells in vitro and inhibits tumor growth in vivo. **a**, **b** Cell viability was determined using CCK8 assay kit 24 h (left) or 48 h (right) after CBD treatment. Data are expressed as the percentage of cell viability compared to control. Statistical differences of HCT116 p53^−/−^ was evaluated by compared to HCT116 p53wt (*p ≤ 0.05, **p ≤ 0.01 and ***p ≤ 0.001) and LS174T compared to SW480 (^#^*p* ≤ 0.05, ^##^*p* ≤ 0.01 and ^###^*p* ≤ 0.001). Two-way ANOVA was used for analysis. Results represent the mean values of three independent experiments (*n* = 3). **c** Schematic diagram of workflow for data in vivo. **d**–**g** HCT116 cells were subcutaneously implanted into SCID mice which received vehicle alone or CBD (20 mg/kg) intraperitoneally 5 times per week over 5 weeks. Tumor size (**d**, **e**) were measured twice per week. **f** Representative examples of tumors treated either with vehicle or CBD are illustrated. **g** The hematoxylin and eosin (H&E) stained sections for observation of morphological changes in cells and immunohistochemistry of mice tumor tissue for detection of cleaved-caspase 3 to identify apoptotic cells directly after CBD treatment (Scale bar: 100 μm). **h** Immunoblot analysis of cytosolic PARP1 and cleaved-PARP1 expression in HCT116 p53wt and HCT116 p53^−/−^ cells 24 h after CBD treatment. **i** TUNEL assay has been performed to detect apoptotic cells after CBD treatment using a colorimetric TUNEL system. *t-*Test was used. Statistical differences of each group were evaluated by comparing the control (*p ≤ 0.05, **p ≤ 0.01 and ***p ≤ 0.001) or to the other group (^#^*p* ≤ 0.05, ^##^*p* ≤ 0.01 and ^###^*p* ≤ 0.001). All data are expressed as the mean ± SD of three independent experiments.

The viability of HCT116 p53wt, HCT116 p53^−/−^, SW480 p53mut, LS174T p53wt cells after treatment with CBD (5–20 µM), as determined using the CCK-8 assay, was reduced in all cell lines in a dose-dependent manner after a 24 h (Fig. 2a) and 48 h (Fig. 2b) incubation. However, the sensitivity of cells to CBD was cell type dependent: HCT116 p53wt (IC50 = 14.67 µM; 95% CI 13.42–15.79), HCT116 p53^−/−^ (IC50 = 24.26 µM; 95% CI 21.33–34.88), LS174T p53wt (IC50 = 7.918 µM; 95% CI 7.039–8.867) and SW480 p53mut (IC50 = 16.58 µM; 95% CI 14.73–19.17) cells. The data revealed that p53 deficient (HCT116 p53^−/−^) and p53 mutant (SW480 p53mut) cells are less sensitive to a CBD treatment than p53 wild-type cells when compared to each other (HCT116 p53wt vs. HCT116 p53^−/−^, LS174T p53wt vs. SW480) (Fig. 2a). However, a significant difference between HCT116 p53wt and SW480 p53mut was observed only with a CBD concentration of 20 µM and an incubation period of 24 h (*p* < 0.0001) (Fig. 2a, b). Unlike p53 deletion (HCT116 p53^−/−^), the two-point mutations (R273H/P309S) in p53 (SW480) triggers a diverse sensitivity to CBD which is probably due to a partially retained p53 function [17]. Based on the IC50 values, a concentration of 15 µM was used as the maximum concentration for in vitro experiments with HCT116 p53wt and p53−/− cells, while LS174T and SW480 cells were treated with 20 µM CBD to enable a better pairwise comparison of intercellular responses to CBD.

We further investigated the antitumoral effect of CBD in a xenograft tumor mouse model. For this, HCT116 p53wt and HCT116 p53^−/−^ cells were injected subcutaneously (s.c.) into immunodeficient SCID mice following a whole-body irradiation with 3 Gy. To avoid methanol induced toxicity in mice, DMSO instead of methanol was used as the vehicle for CBD in the mouse experiment. We could demonstrate that irrespective of the vehicle (methanol or DMSO), CBD exerts comparable effects on cell viability of HCT116 p53wt cells, as demonstrated in a CCK8 assay (Supplementary Fig. 1a). Tumor-bearing mice were treated five times a week with CBD with the non-toxic dose of 20 mg/kg for 5 weeks as illustrated schematically in Fig. 2c. A significant reduction in the volume of HCT116 p53wt tumors was observed in mice after CBD treatment compared to the DMSO vehicle control from day 37 onwards (Fig. 2d). In contrast, the volume of HCT116 p53^−/−^ tumors was not significantly reduced by the CBD treatment at any time point (Fig. 2e), suggesting that a long-term CBD treatment only decreases the growth of p53wt, but not p53^−/−^ tumors. Preliminary studies demonstrated that the growth of HCT116 p53wt and HCT116 p53^−/−^ cell-derived tumors was comparable when 2.0 × 10^6^ p53wt and 2.8 × 10^6^ p53^−/−^ cells were injected s.c., respectively (Supplementary Fig. 1b). The treatment with CBD or the vehicle DMSO did not elicit any negative side effects in mice since the body weight of the mice was comparable in all treatment groups over a period of 40 days (Supplementary Fig. 1c). Representative examples of tumors treated either with vehicle or CBD are illustrated in Fig. 2f. Immunohistochemical analysis revealed a cell vacuolization in H&E sections of HCT116 p53wt tumors following CBD treatment, which was barely seen in HCT116 p53^−/−^ tumors (Fig. 2g). Moreover, CBD induced a weak elevation in caspase-3 expression in HCT116 p53wt tumors which is indicative for apoptosis, whereas almost no apoptosis was apparent in HCT116 p53^−/−^ tumors (Fig. 2g). With respect to DNA strand breaks, cleaved PARP1 was only accumulated in HCT116 p53wt cells upon a treatment with CBD at a concentration of 15 µM. This finding corresponds to an increase in the proportion of TUNEL positive cells (Fig. 2h, i). Similar to HCT116 p53^−/−^ cells, there was no significant difference in cleaved PARP1 expression after CBD treatment in SW480 p53mut cells (Fig. 2h and Supplementary Fig. 2a). However, in contrast to HCT116 p53wt cells, in LS174T p53wt cells neither cleaved PARP1 nor cleaved-caspase-3 were found to be upregulated (Supplementary Fig. 2a, b).

### CBD induces a G0/G1 cell cycle arrest and a p53-dependent over production of ROS

**Fig. 3:**
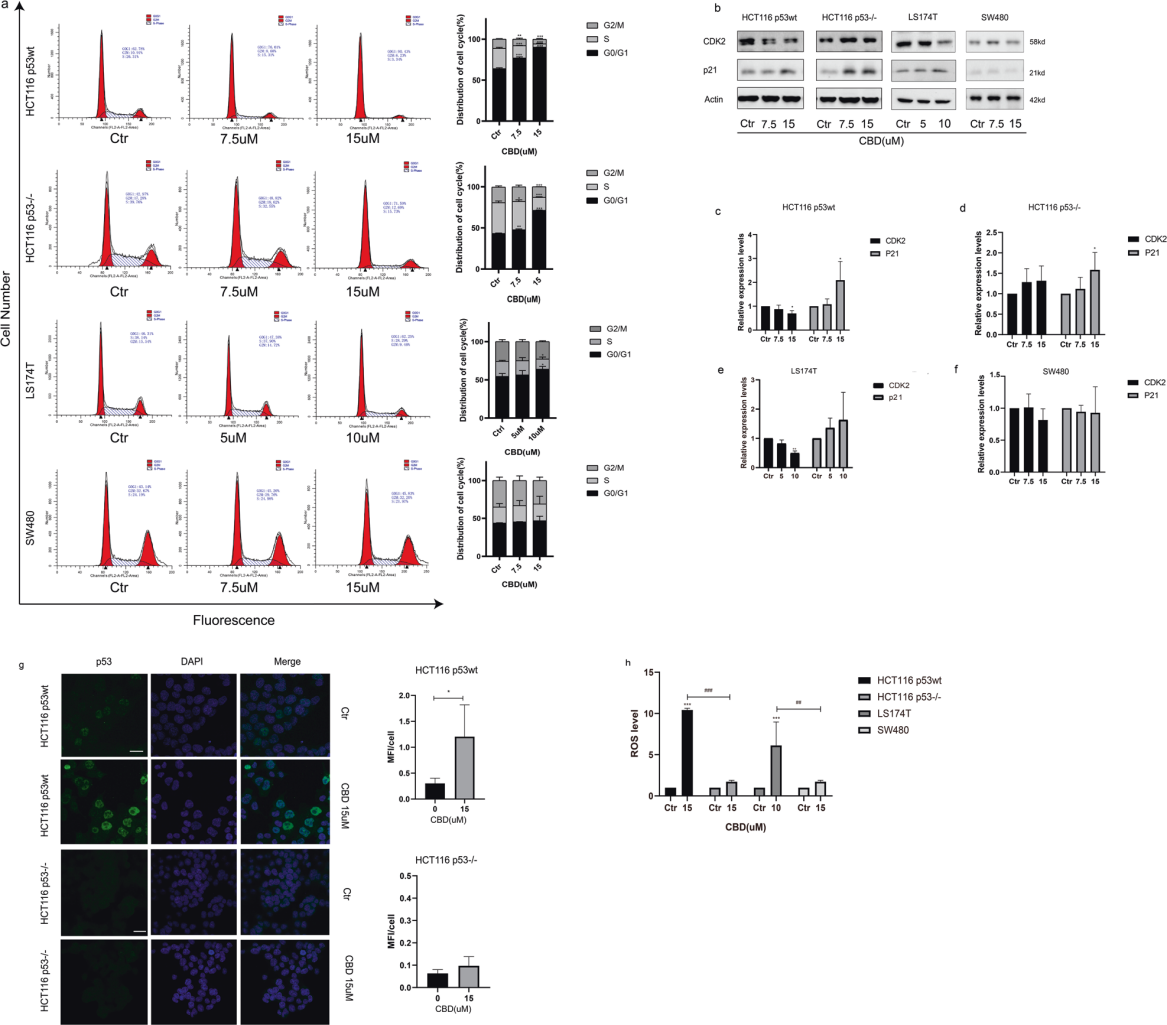
CBD induces G0/G1 cell cycle arrest. **a** Flow cytometry was used to determine the cell cycle distribution in CBD-treated CRC cells. The quantification of each cell cycle phase is shown in the adjacent bar chart. **b** The expression levels of CDK2 and p21 protein in CBD-treated CRC cells were detected by Western blotting. **c**–**f** The ratio of protein levels was normalized to the values of the control. **g** HCT116 p53wt and p53^−/−^ cells stained for the expression of p53 were analyzed by confocal microscopy (Scale bar: 25 μm). Quantification of mean fluorescence intensity per cell was presented as adjacent bar charts. p53(green). Nuclei, DAPI (blue). Statistical differences of each group were evaluated by compared to the control group (*p ≤ 0.05, **p ≤ 0.01 and ***p ≤ 0.001). *t*-Test was used. Results are shown as mean ± SEM (HCT116 p53wt *n* = 36–43, HCT116 p53−/− *n* = 53–72). **h** After a 24 h CBD treatment, intracellular reactive oxygen species (ROS) levels were determined using the DCFDA assay. One-way ANOVA analysis was used. Statistical differences of each group were evaluated by comparing the control (*p ≤ 0.05, **p ≤ 0.01 and ***p ≤ 0.001) or to the other group (^#^*p* ≤ 0.05, ^##^*p* ≤ 0.01 and ^###^*p* ≤ 0.001). All data are expressed as the mean ± SD of three independent experiments.

CDK2 kinase plays a crucial role in regulating the G1-S transition. Mitotic cell arrest occurs when CDK2 kinase activity is inhibited by the CDK-specific inhibitor p21 [18]. We found that CBD in a concentration range of 7.5 and 15 µM induces a significant G0/G1 phase arrest which was more pronounced in p53wt (Fig. 3a) cells than p53^−/−^ or p53mut cells, concomitant with a p53-dependent CDK2 downregulation and a p53-independent p21 upregulation (Fig. 3b–d). A similar trend was found in another LS174T p53wt cells (Fig. 3e). As expected, CBD had no significant impact on the expression of cell cycle proteins in SW480 p53mut cells (Fig. 3f). p53 is a transcription factor that acts as an upstream regulator for reactive oxygen species (ROS) in response to stress by activating or repressing several ROS-regulating genes, such as glutathione peroxidase (GPX) and p53-induced genes (PIGs) [19] which act in a pro- or an antioxidative environment. Our results indicate that the significant increase in ROS production induced by CBD is proportional to the p53 functional activity, since a nuclear translocation of p53 (Fig. 3g, h) together with a ROS accumulation following CBD treatment only occurs in p53wt cells (Fig. 3h), but not in p53 deficient cells (Fig. 3h).

### Heat shock protein 70 (Hsp70) attenuates CBD-induced apoptosis by inhibiting the p53-dependent caspase-8/9/3 pathway

**Fig. 4:**
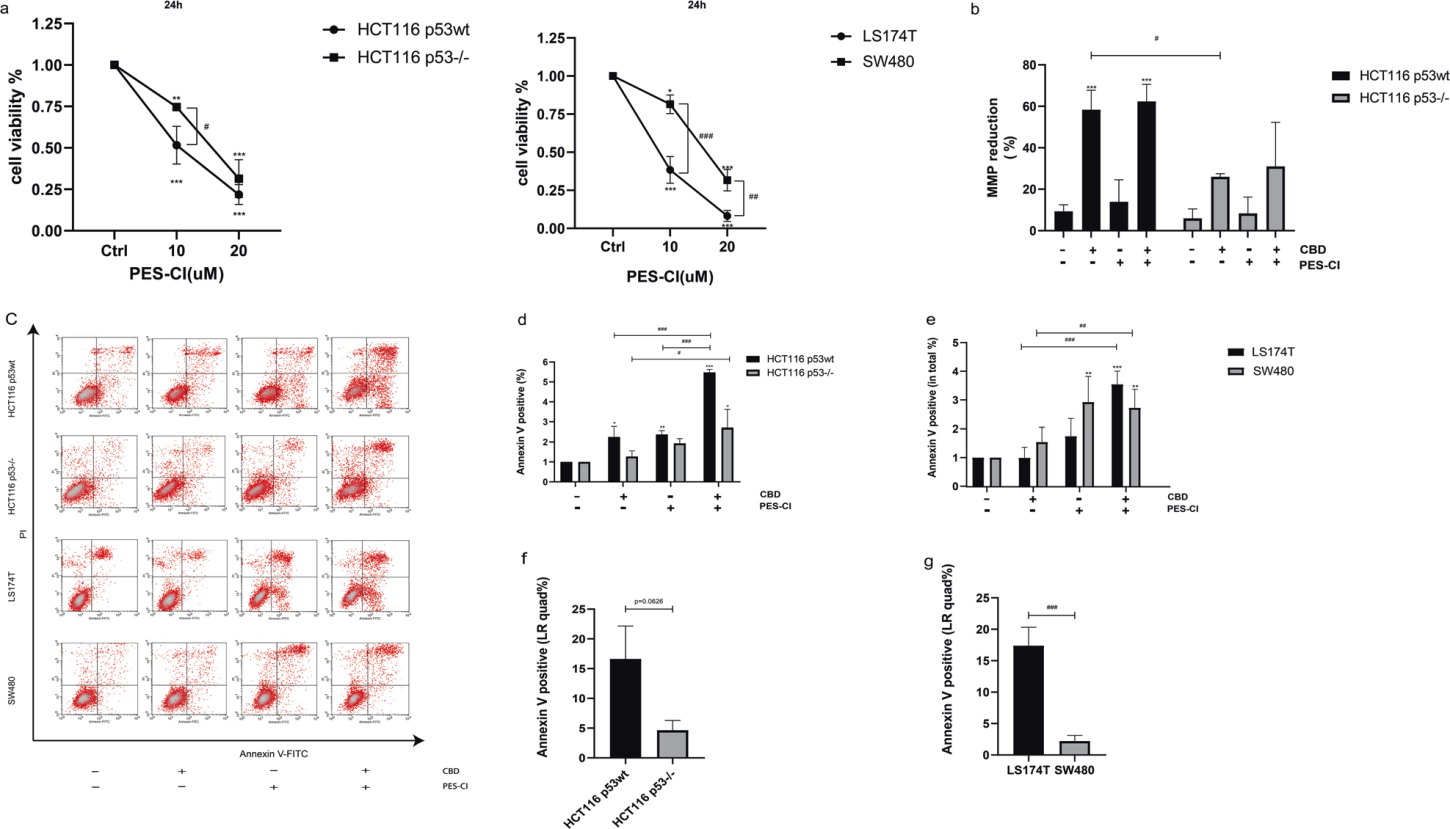
The Hsp70 inhibitor PES-CI potentiates the antitumor effect of CBD. **a** Viability of CRC cells treated with PES-CI. **b** CBD-induced reduction in mitochondrial membrane potential (MMP) (%) in HCT116 cells. **c** Apoptosis determined by Annexin V/PI staining. **d**, **e** Percentage of apoptotic CRCs (Annexin V positive: UR quad% + LR quad%). **f**, **g** Percentage of early apoptotic Annexin V positively stained CRCs (LR quad%). Statistical differences of each group were evaluated by comparing the control (*p ≤ 0.05, **p ≤ 0.01 and ***p ≤ 0.001) or to the other group (^#^*p* ≤ 0.05, ^##^*p* ≤ 0.01 and ^###^*p* ≤ 0.001). One-way ANOVA, two-way ANOVA analysis or *t*-test was used. All data are expressed as the mean ± SD of three independent experiments.

**Fig. 5:**
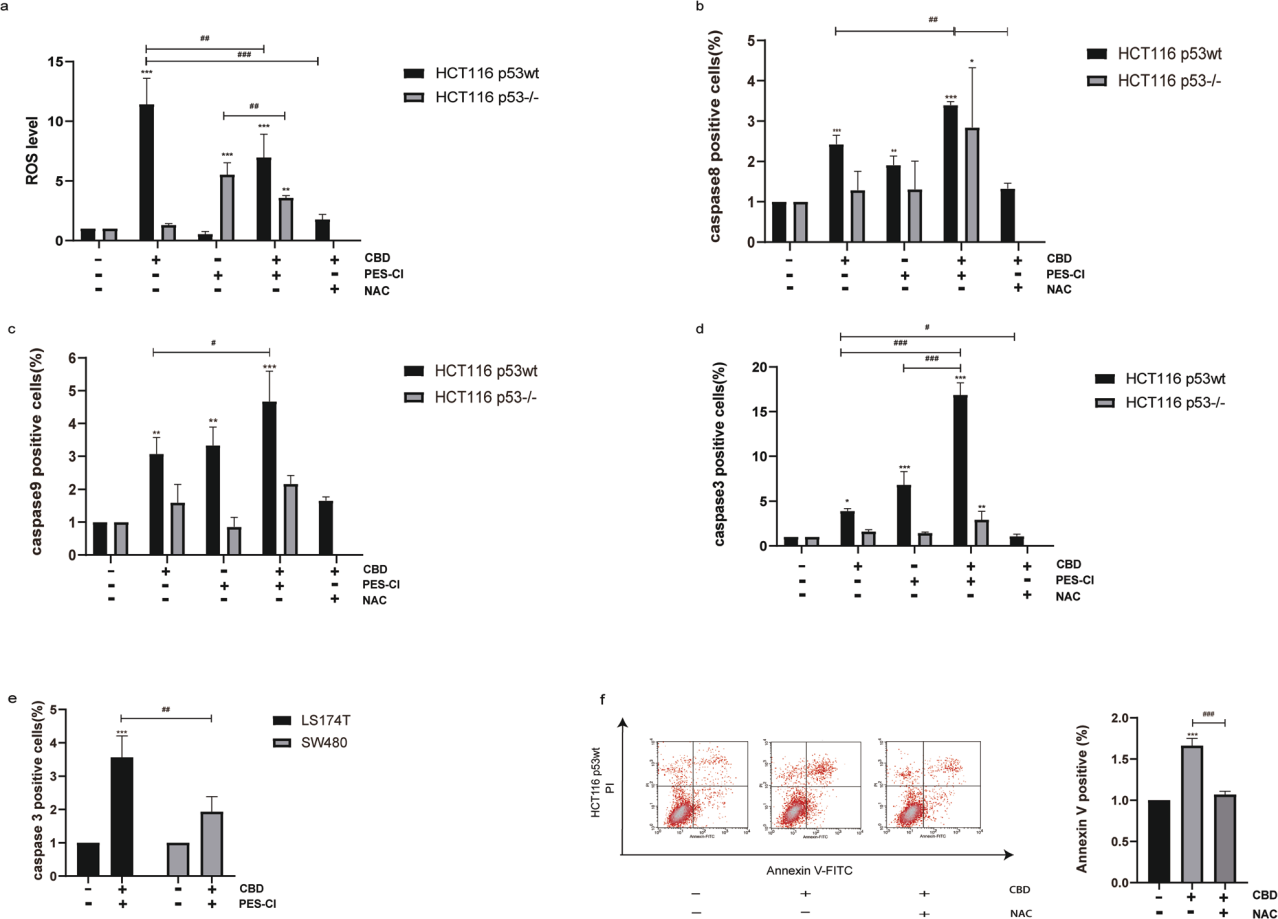
Hsp70 inhibition enhances the p53-dependent cleaved caspase-8/9/3 pathway. **a** Flow cytometry was used to monitor ROS generation in both p53wt and p53^−/−^ HCT116 cells by staining with DCF-DA after CBD (15 µM) treatment in combination with PES-CI (IC50 value accordingly) or ROS scavenger NAC (2.5 mM). **b**–**d** Cleaved caspase-8/9/3 levels as measured by flow cytometry. **e** The p53-dependent activation of caspase-3 in LS174T and SW480 cells after treatment with CBD and PES-CI. **f** NAC attenuated apoptosis induced by CBD in HCT116 p53wt cells. Statistical differences of each group were evaluated by comparing the control (*p ≤ 0.05, **p ≤ 0.01 and ***p ≤ 0.001) or to the other group (^#^*p* ≤ 0.05, ^##^*p* ≤ 0.01 and ^###^*p* ≤ 0.001). One-way ANOVA, two-way ANOVA or *t*-test was used. All data are expressed as the mean ± SD of three independent experiments.

Hsp70, a member of the HSP70 family encoded by HSPA1, is very strongly upregulated by heat stress and a large variety of other stress stimuli including toxic chemicals, particularly heavy metals such as arsenite, cadmium, copper, mercury, etc. Heat shock proteins were originally discovered by Ferruccio Ritossa in the 1960s when a lab worker accidentally boosted the incubation temperature of Drosophila (fruit flies). When examining the chromosomes, Ritossa found a “puffing pattern” that indicated the elevated gene transcription of an unknown protein [6, 7]. This was later described as the “Heat Shock Response” and the corresponding proteins were termed as “Heat Shock Proteins” (HSPs). It is well accepted that many tumor cells overexpress Hsp70 and that this overexpression correlates with resistance to apoptosis-inducing agents, whereas a downregulation of Hsp70 results in an increased sensitivity toward these agents [20]. Hsp70 can interfere with apoptosis pathways by blocking the aggregation of the functional apoptosome [21]. PES-CI, an Hsp70 inhibitor, binds to the substrate domain of Hsp70 which is important for the binding of client proteins, and thereby represses the enzymatic activity of the anaphase-promoting complex/cyclosome (APC/C) [22]. Previous study showed that PES interacts with the major stress-inducible Hsp70, but not with the constitutively expressed Hsc70 [23]. To better understand the potential role of Hsp70 on the antitumoral effects of CBD, we therefore interrogated the potential interaction partners of Hsp70 using the PES-CI inhibitor. As expected, PES-CI reduces the viability of all CRC cells in a dose-dependent manner (Fig. 4a), with the sensitivity of HCT116 p53wt (IC_50_ = 10.33 µM; 95% CI 4.937–13.18), HCT116 p53^−/−^ (IC_50_ = 14.96 µM; 95% CI 12.59–17.91), LS174T (IC_50_ = 8.451 µM; (95% CI 5.201–9.902) and SW480 (IC_50_ = 15.78 µM; 95% CI 13.93–17.86) to PES-IC depending on the p53wt status. A co-treatment of CRC cells with the PES-CI and CBD results in distinct morphological changes which was more pronounced in p53wt cells. In general, the cells became round, detached from the surface of the culture flask and underwent apoptosis (Supplementary Fig. 3) which was documented by a disruption of the mitochondrial membrane potential (Fig. 4b), concomitant with a substantial increase in the proportion of Annexin V/PI double positive, apoptotic cells (Fig. 4c–g). In contrast, the population of Annexin-V single positive cells was only significantly enhanced in p53wt cells, but not in p53^−/−^ or p53mut CRC cells (Fig. 4c–g). We further determined cleaved caspase-8/9/3 by flow cytometry to confirm apoptosis induction in wild-type p53 CRC cells. We demonstrated that CBD treatment alone considerably increased the levels of reactive oxygen species (ROS), but had only a moderate effect on the percentage of cells positive for Annexin-V and cleaved caspase-8/9/3 (Fig. 5a–d). However, when CBD was used in combination with the Hsp70 inhibitor PES-CI, the expression of cleaved caspase-9/3 was significantly elevated in cells harboring wild-type p53 (HCT116 p53wt, LS174T p53wt), but not in p53 deficient or p53 mutant cells (HCT116 p53^−/−^ and SW480 p53mut) (Fig. 5d, e). Caspase-8 displays a pivotal role in the p53-independent apoptotic signaling pathway following CBD treatment, and Hsp70 inhibition significantly accelerated caspase-8 dependent apoptosis (Fig. 5b). As discussed previously [24, 25], the antioxidant N-acetyl cysteine (NAC) attenuates the antitumoral effect of CBD, as demonstrated by a reduction in the proportion of Annexin-V positive cells and the levels of cleaved caspase-8 (*p* = 0.0060), cleaved caspase-9 (*p* = 0.0545), cleaved caspase-3 (*p* = 0.0215) in HCT116 p53wt cells (Fig. 5).

### Protective macroautophagy induced by CBD is related to ROS accumulation and wild-type p53 acts as a potential upstream regulator

Associated with the heat shock (stress protein) system, macroautophagy is a regulatory mechanism which maintains cellular protein homeostasis by sequestering and transporting large protein aggregates and damaged or senescent organelles to lysosomes for degradation.

**Fig. 6:**
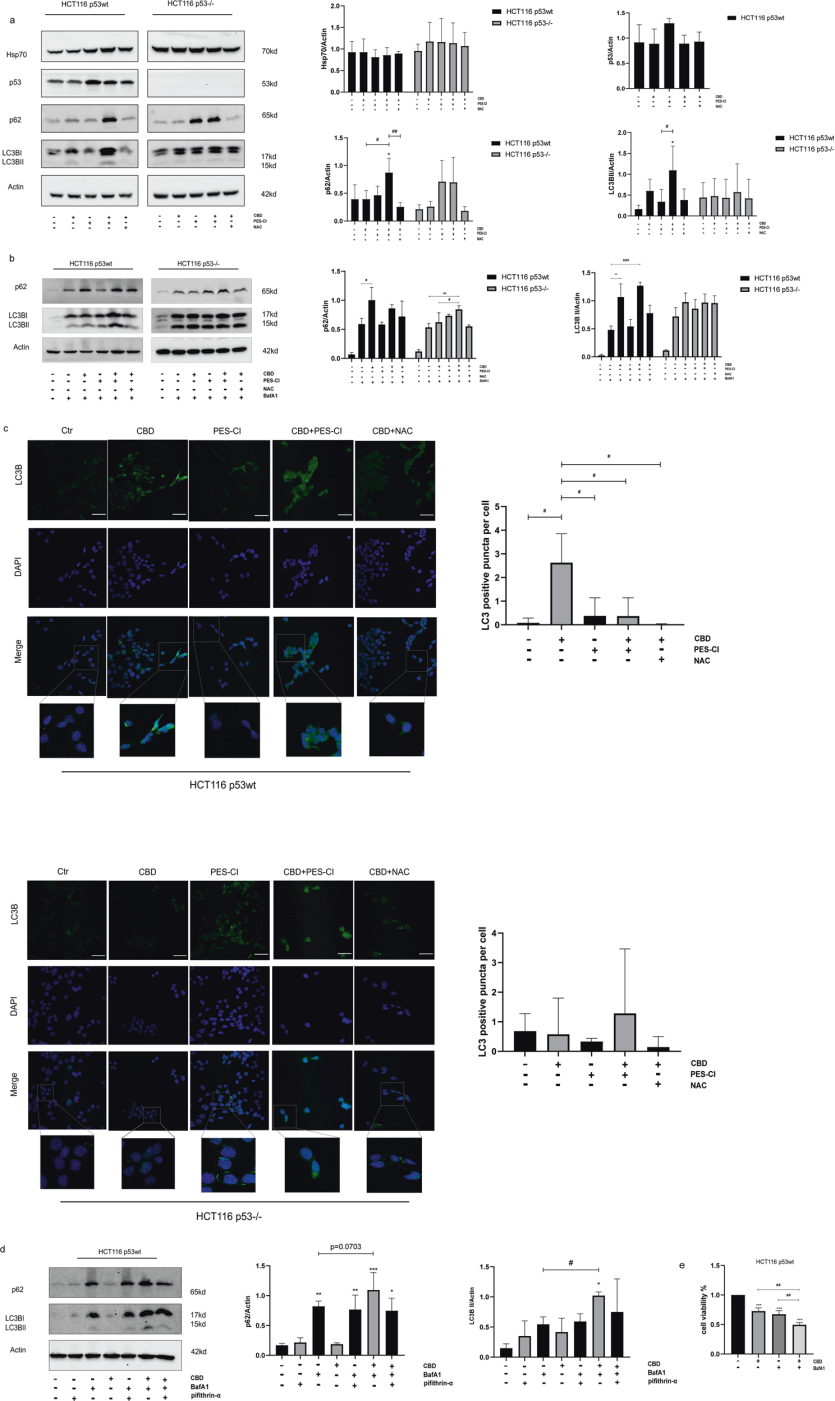
Protective autophagy induced by elevated ROS levels after CBD treatment in HCT116 p53wt cells. **a** Immunoblot of cytosolic Hsp70, p62 and LC3BII expression levels in HCT116 p53wt and HCT116 p53^−/−^ cells after CBD treatment (15 µΜ) and a co-treatment with the Hsp70 inhibitor PES-CI (IC_50_ value correspondingly) and/or the ROS scavenger NAC (2.5 mM). Adjacent bar charts show the quantification of Hsp70, p62 and LC3BII expression upon a combined treatment with the different reagents in HCT116 p53wt and p53^−/−^ cells. **b** Representative immunoblot showing the expression of intracellular p62 and LC3B 24 h after co-incubation with BafA1 (50 nM), a quantification of the p62 and LC3BII expression level are shown in the adjacent bar chart. **c** Quantification of LC3B vesicles using confocal fluorescence microscopy. (Left) Exemplary confocal images of LC3B (green) expressing HCT116 p53wt and HCT116 p53^−/−^ cells. Nuclei, DAPI (blue). Scale bar: 100 µm. (Right) Quantification of the LC3B vesicles per cell of HCT116 cells. Statistical differences of each group were evaluated by compared to the other group (^#^*p* ≤ 0.05, ^##^*p* ≤ 0.01 and ^###^*p* ≤ 0.001). One-way ANOVA was used. Results are shown as mean ± SEM (HCT116 p53wt *n* = 20–85, HCT116 p53^−/−^ *n* = 9–80). **d** The amount of p62 and LC3B expression in CBD-treated HCT116 p53wt cells with or without p53 inhibitor pifithrin-α (20 uM) and BarfA1. The relative expression levels of p62 and LC3B are shown in the adjacent bar graph. **e** CBD-induced cytoprotective autophagy is associated with a reduced cell viability following co-treatment with BafA1, as determined with the CCK-8 viability assay in HCT116 p53wt cells. Statistical differences of each group were evaluated by comparing the control (*p ≤ 0.05, **p ≤ 0.01 and ***p ≤ 0.001) or to the other group (^#^*p* ≤ 0.05, ^##^*p* ≤ 0.01 and ^###^*p* ≤ 0.001). One-way ANOVA was used. All data are expressed as the mean ± SD of at least three biological replicates.

Microtubule-associated protein 1A/1B-light chain 3 (LC3) is a ubiquitously distributed soluble protein which is incorporated into the expanded phagosome upon binding to phosphatidylethanolamine [26]. In this process, phagosome-bound LC3BII acts as a pillar for proteins bound to ubiquitinated substrates [27]. During the macroautophagy flux, autophagosomes fuse with lysosomes, which subsequently hydrolyze and degrade the content inside a cell [26]. The ability of PES-Cl to inhibit macroautophagy has been documented in several different autophagy assays [22, 28]. As shown in Fig. 6a, LC3BII appeared to be elevated in HCT116 p53wt cells, but not in HCT116 p53^−/−^ cells. p62 behaves as a linker in the macroautophagy process, and its aberrant aggregation indicates an impairment of the autophagy degradation pathway. To distinguish whether the accumulation of these proteins is caused by an increased macroautophagy or by a dysregulated auto-lysosomal degradation, we examined the levels of LC3BI and LC3BII in HCT116 p53wt cells 24 h after exposure to CBD in the presence and absence of BafA1 (50 nM), an autophagy inhibitor which halts the autophagic flux by inhibiting late-stage fusion between autophagosomes and lysosomes [29]. A co-incubation with BafA1 significantly enhances the CBD-induced increase of these proteins in HCT116 p53wt cells (Fig. 6b), which means that CBD accelerates the macroautophagy process. Similar results were shown in Fig. 6c. The number of LC3B vesicles in p53wt cells were significantly increased after CBD treatment, while they did not significantly alter in p53 deficient cells. In line with a previous report [28], a 24 h co-incubation with CBD (15 µM) and PES-CI prevented the autophagy flux in both HCT116p53wt and HCT116 p53^-/-^cells, whereas p62 overexpression was primarily dependent on the Hsp70 inhibitor in PES-CI in HCT116 p53^−/−^ cells. Additionally, NAC slightly suppressed the CBD-induced macroautophagy, which was induced by ROS in HCT116 p53wt cells, as it attenuated the p62 and LC3BII expression as well as LC3B vesicles without BafA1 (*p* = 0.3294 and *p* = 0.3821, respectively; Fig. 6a, c) or with BafA1 (*p* = 0.2118 and *p* = 0.1422, respectively; Fig. 6b). Autophagy was once known to be triggered by the lack of p53 or mutant p53 mostly during the G1 phase and to a lesser extent in the S phase to avoid that cells enter the G2/M phase [30]. However, our study found that the p53 inhibitor pifithrin-α (20 µM) decelerates the cytosolic expression of p62 (*p* = 0.026) and LC3BII (*p* = 0.2155) which induced by CBD after a 24 h cotreatment with BafA1 (Fig. 6d). Based on the results of the viability assay, this kind of autophagy displays a cytoprotective effect (Fig. 6e). In summary, our data indicates that CBD stimulates protective macroautophagy partially though ROS accumulation and it is likely that wild-type p53 displays a role in this process as an upstream regulating factor.

### p53-associated ROS accumulation activates the Nrf2 pathway

**Fig. 7:**
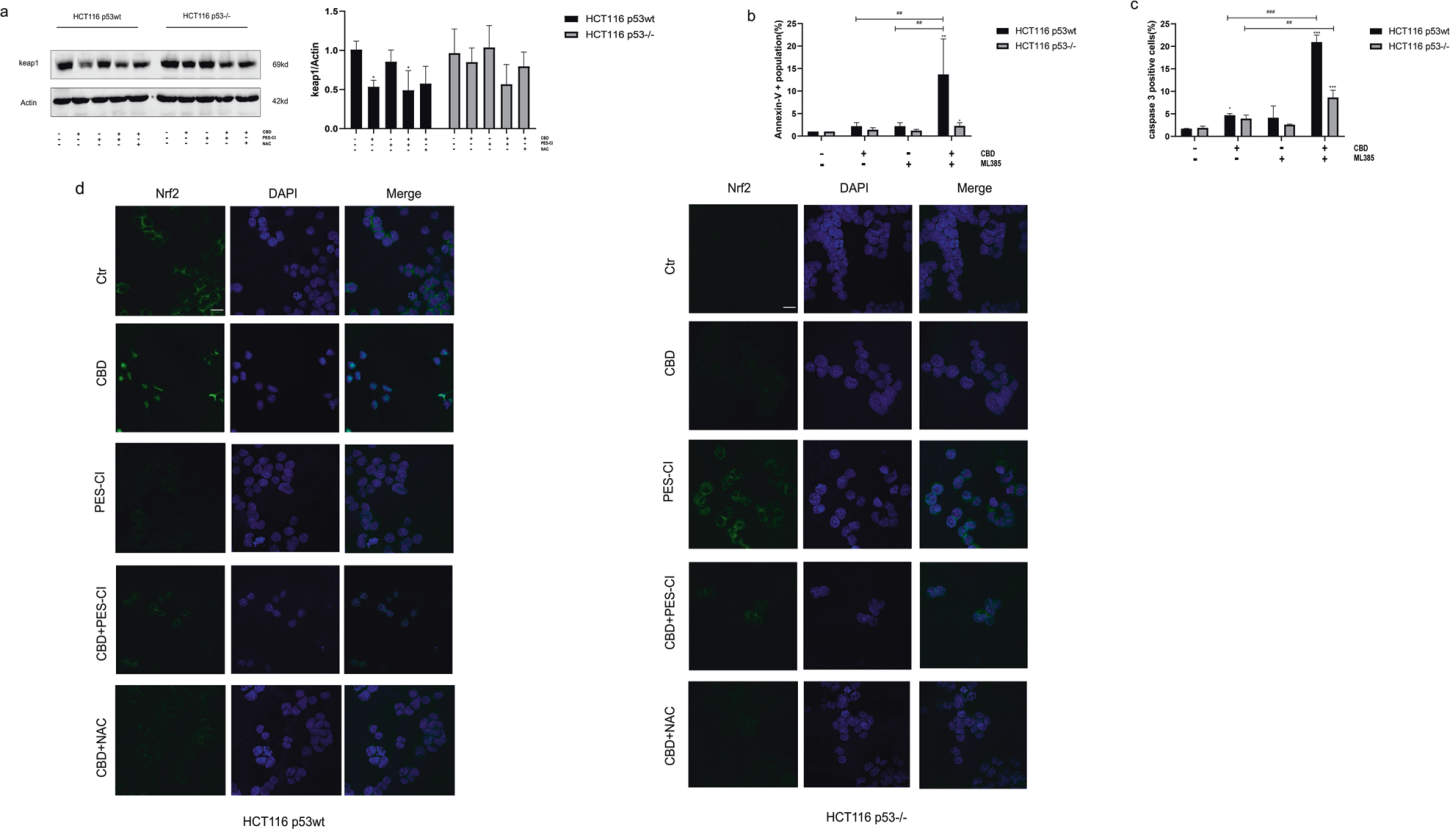
p53-associated oxidative stress activates the keap1-Nrf2 pathway. **a** Immunoblot of keap1 and Actin. A quantification of the keap1: actin ratio is shown in the adjacent bar chart. **b**, **c** Inhibition of Nrf2 by ML385 (5 uM, 24 h) enhances the antitumoral effect of CBD via an increase in apoptosis in p53wt cells, whereas only a moderate effect is observed for p53 knockout cells. Statistical differences of each group were evaluated by comparing the control (*p ≤ 0.05, **p ≤ 0.01 and ***p ≤ 0.001) or to the other group (^#^*p* ≤ 0.05, ^##^*p* ≤ 0.01 and ^###^*p* ≤ 0.001). One-way ANOVA was used. All data are representative of three independent experiments. **d** HCT116 p53wt and p53^−/−^ cells stained for Nrf2 after different treatments as determined by confocal microscopy (Scale bars: 25 μm).

The macroautophagy element p62 is a target gene of the Nrf2 (nuclear factor erythroid-derived 2-like 2-antioxidant response element, ARE) transcriptional pathway and its accumulation has been reported to trigger autophagic degradation of the Nrf2 inhibitory protein keap1 (kelch-like ECH-associated protein 1) thereby sustaining the activation of Nrf2. [31]. In our study, overexpression of p62 and macroautophagy activation were associated with a downregulation of keap1 during oxidative stress which results in the nuclear translocation of Nrf2 (Fig. 7a, d) in HCT116 p53wt cells. Since this effect can be reversed by NAC (Figs. 6a and 7d) it is assumed that p53wt-associated oxidative stress continuously triggers the autophagic degradation of keap1 and thereby contributes to the excessive activation of Nrf2. In addition to the degradation of keap1 which is induced by an activated autophagy, inhibition of autophagy also leads to a cytoplasmic accumulation of p62 and a persistent activation of Nrf2 [32]. The inhibition of autophagy by the Hsp70 inhibitor PES-CI results in a decrease in reactive oxygen species (ROS) (Fig. 4a) and a corresponding decrease in the keap-1 expression in both p53^−/−^ and p53wt HCT116 cells (*p* = 0.3415, *p* = 0.0276, respectively) (Fig. 7a). However, the nuclear translocation of Nrf2 was attenuated only in HCT116 p53wt cells (Fig. 7d). This effect might be associated with a repression of Hsp70-assisted Nrf2 nuclear translocation. However, Nrf2 overexpression occurs only after a 24 h treatment with the Hsp70 inhibitor PES-CI in p53 deficient cells and is associated with an increased ROS production (Figs. 5a and 7d). Recent study has also demonstrated that p62 is a potential target of Nrf2 [33], and that the induction of the p62 gene by oxidative stress is mediated via Nrf2. As shown in Fig. 7d, PES-CI pronounced the Nrf2 expression in HCT116 p53^−/−^ cells, which might be an explanation for the abnormal accumulation of p62 after a treatment with PES-CI (Fig. 6a). NAC partially reverses the downregulation of keap1, but almost completely suppresses the expression of Nrf2 (Fig. 7a, d) in HCT116 p53wt cells. A Nrf2 inhibitor (ML385, 5 µM) enhances the antitumor effect of CBD by increasing apoptosis in p53wt cells, whereas there is a blunted induction of apoptosis in p53 knockout cells (Fig. 7b, c). Activation of the keap1-Nrf2 system is supposed to protect cells from excessive ROS toxicity, which is caused by CBD, as suggested by the oxidative stress hypothesis [34] and thereby inhibits the activation of the apoptotic pathway, mediated by p53.

## Discussion

In response to a wide range of stimuli that might cause genomic instability, the tumor suppressor protein p53 acts as a redox-active transcription factor that coordinates and controls cellular responses to maintain genomic integrity [35]. Following transcriptional activation of p53, for example, p21 expression is upregulated which results in RB-E2F complex formation and downregulation of a large number of cell cycle genes [36]. Double allelic mutations in the TP53 gene locus result in the loss of wild-type function of p53 [4]. We found that the cell cycle arrest induced by CBD is associated with an upregulated p21 expression which is p53 independent. It is known that missense mutations in the DNA-binding domain of p53 partially or completely lose their tumor suppressive capability and enhance invasion, migration and treatment resistance of tumor cells [5, 37]. Reactive oxygen species (ROS) can act as signaling molecules or as cellular toxins. Wild-type p53 organizes the transcription of multiple genes in response to cellular stressors that cause DNA damage, minimize the dissemination of damaged DNA [38] by inducing cell cycle arrest, senescence, or apoptosis through differential activation of different target genes [39]. Our study suggests that the CBD-induced ROS production depends on a functional activation of p53, since the ROS accumulation and nuclear translocation of p53 only occurs in p53 WT cells upon CBD treatment (Fig. 3). Recent studies showed that ROS performs dual activities as an up-stream signal triggering p53 activation and a downstream factor mediating apoptosis. The repression of antioxidant genes and transactivation of pro-oxidant enzymes by a p53 activation at the promoter level have been identified as an additional way to increase oxidative stress [40]. p53 directly regulates glycolysis and apoptosis regulator (TIGAR) and cytochrome c oxidase 2 (sCO2) gene expression, for instance, thereby enhancing oxidative phosphorylation and overexpression in ROS accumulation, which play major roles in programmed cell death [41–43]. As shown in Figs. 3 and 4, apoptotic pathways were only moderately activated (cleaved caspase-8/9/3 upregulated) by CBD in the case of a dramatic increase over ROS, and the antioxidative ROS scavenger NAC, further diminished the p53-dependent apoptosis.

It is well accepted that Hsp70, which is abundantly overexpressed in many different cancer types, suppresses both the extrinsic and intrinsic apoptotic pathway and thereby allows cancer progression [44]. Transcription factor Jun is a protein encoded by the JUN gene. c-Jun, in combination with protein c-Fos, forms the AP-1 early response transcription factor, which mediates cell cycle progression and anti-apoptotic activity [45, 46]. JNK (c-Jun-N-terminal Kinase) activity was shown to play an important role in the induction of the intrinsic apoptotic pathway through mitochondrial dysfunction [47] that can be inhibited by the Hsp70-CHIP complex [48], and wild-type p53 mediates JNK-dependent apoptosis [49]. The results of our study revealed that the Hsp70 inhibitor PES-CI enhances the intrinsic apoptotic pathway triggered by CBD in HCT116 p53wt cells, as illustrated in Fig. 4, which is associated with a considerable overproduction of cleaved caspases-9/3 [50]. In addition, the cysteine protease caspase-8, which represents the extrinsic apoptotic pathway [51], was activated by CBD in a p53-independent manner (Fig. 4). Based on our transcriptional process enrichment analysis (Fig. 1), the transcription factor JUN signaling pathway is one of a series of CBD-CRC-relative transcriptional processes, which might provide an up-stream signal of p53 mediated cell death upon CBD treatment.

ROS-induced DNA damage activates the PARP1 signaling pathway which further initiates autophagy [52]. A specialized autophagy response, resulting in the removal of damaged organelles and protecting cells, can be triggered by organellar stress [53]. We found an increased production of reactive oxygen species induced by CBD and subsequently an activated protective macroautophagy, which might hinder the programmed cell death mediated by p53. This protective macroautophagy can be reversed by an Hsp70 inhibition that blocks autophagy. A previous study showed that PES interacts with Hsp70, but not with Hsc70 [23]. However, it was later proven that PES inhibited both Hsp70 and Hsc70 in vitro [54]. PES-CI, which is derived from PES containing 2-(3-chlorophenyl) ethynesulfonamide, has superior ability to inhibit macroautophagy compared to PES [22]. At present, the available evidence is insufficient to rule out the possibility that the ability of PES-Cl to inhibit autophagy is partially due to a suppression in Hsc70-mediated CMA (chaperone-mediated autophagy) [55]. This possibility and the potential function of CMA regarding the antitumor effect of CBD remains to be explored. A mildly decelerated p62 induced by a p53 inhibitor suggests a potential mutual constraint relationship between these two signaling pathways. Similar results were obtained by in vitro and in vivo analysis, showing a weak upregulation of cleaved caspase-3 upon CBD treatment in wild-type cells despite an intense ROS generation (Figs. 2–4). The protective role of Hsp70 was partially dependent on macroautophagy activation, indicating a crosstalk between Hsp70 and macroautophagy in the oxidative stress response induced by CBD.

Autophagy is one of the main routes to eliminate damaged material induced by oxidative stress. Oxidative stress is associated with elevated levels of intracellular reactive oxygen species (ROS) that trigger the activation of transcription factors, such as Nrf2, to maintain redox homeostasis by inducing the antioxidative pathways [56]. In this study we could show that CBD causes the production of ROS which in turn activates the keap1-Nrf2-antioxidant system to attenuate ROS toxicity (Fig. 7). In addition, the enhanced macroautophagy induced by keap1 ablation can be suppressed by an Hsp70 inhibition which results in an downregulation of keap1 but a weaker translocation of Nrf2 into the nucleus (Fig. 7), thereby eliminating the anti-apoptotic effect This attenuated nuclear translocation of Nrf2 may be related to the chaperone role of Hsp70 [57], which maintains intracellular environmental homeostasis after stressor damage by assisting Nrf2 nuclear transport.

## Conclusion

**Fig. 8:**
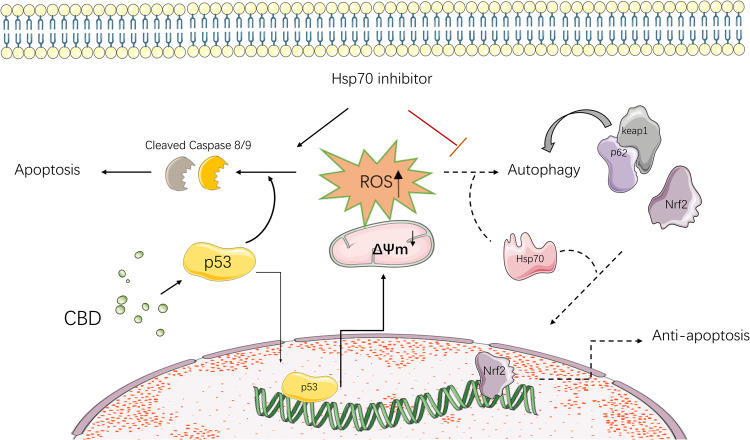
Potential mechanism of CBD in antitumor effect. Both the protective autophagy route and the programmed cell death pathway are activated in response to mitochondrial malfunction and ROS overproduction, which are caused by p53 nuclear translocation after CBD treatment. Hsp70-mediated autophagy degrades keap1, consequently, unbound Nrf2 is released for nuclear translocation and blocks p53-regulated apoptosis.

For a long time, it was considered that the primary mechanism by which TP53 suppresses tumor formation was the production of apoptotic cell death. Herein we show that the p53 status, a main determinant of anti-neoplastic drug efficacy, appears to impact also the cellular oxidative stress after CBD treatment. A CBD treatment induces complex events in p53wt CRC cells, including autophagy, activation of the chaperone system (Hsp70 induction) and stimulation of the keap1-Nrf2 signaling pathway. Inhibition of Hsp70 was shown to shift the CBD-induced autophagy toward caspase-8/9 mediated apoptosis. Taken together a combined treatment consisting of CBD and Hsp70 inhibition may enable an improved programmed tumor cell death in p53wt CRC cells (Fig. 8).

## Methods

### Reagents and treatment

All reagents were purchased from Sigma-Aldrich. Cannabidiol was dissolved in methanol and DMSO. ML385 (keap1-Nrf2 inhibitor), Bafilomycin A1 (specific inhibitor of vacuolar type H+-ATPase, V-ATPase) and PES-CI (Hsp70 inhibitor) were dissolved in DMSO (D2650, Sigma). The antioxidant NAC (N-Acetyl*-*L-cysteine) was diluted in sterile H_2_O. Growth medium was used as the vehicle for test substances, and contained 0.5% (v/v) methanol for cannabidiol and ≤0.1% (v/v) DMSO for ML385, PES-CI and Bafilomycin A1.

### Screening of potential targets of CBD and enrichment assay

Well-reported pharmacological targets of cannabidiol were identified using the Drugbank [58], Swiss Target Prediction [59] and SuperPred [60] databases. The Genecard [61] database was used to screen for pathogenic targets in CRC. Potential targets for CBD in CRC were analyzed using Venn diagrams and the interactome network, functional processes and molecular pathways visualized using Metascape [62].

### Cells and cell culture

The HCT116 (p53wt) was purchased from ATCC and HCT116 p53 double knockout (HCT116 p53^−/−^) human colon adenocarcinoma cell lines was kindly provided by Prof. Bert Vogelstein (Johns Hopkins University, Baltimore, USA) and maintained in McCoy’s 5A medium (Sigma-Aldrich). The SW480 human adenocarcinoma colorectal cancer cell line (ATCC#CCL-228™; ATCC, USA) which carries two-point mutations (R273H/P309S) was cultured in Dulbecco’s modified Eagle’s medium (DMEM, Sigma-Aldrich) supplemented with 10% (v/v) fetal bovine serum (FBS) and 1% (v/v) penicillin/streptomycin (Sigma-Aldrich). LS174T (p53wt, ATCC#CL-188™; ATCC, USA) [63] human adenocarcinoma colorectal cancer cells were cultured in DMEM supplemented with 10% (v/v) FBS and 1% (v/v) penicillin/streptomycin (Sigma-Aldrich), 2 mM L-glutamine (Sigma-Aldrich) and 1 mM sodium pyruvate (Sigma-Aldrich) at 37 °C in a 5% CO_2_ atmosphere. After seeding at the desired density, cells were incubated overnight prior to the experiments. Cells were routinely checked for mycoplasma contamination.

### Cell viability assay

The effect of CBD on the viability of cells was determined using a CCK-8 (Cell Counting Kit-8,) assay (Sigma-Aldrich). For this, cells were incubated with culture medium containing 5, 10, 15, 20 µM of CBD for 24–48 h, after which the absorbance at 450 nm was measured using a PerkinElmer 2030 multilabel reader (PerkinElmer LAS GmbH, Germany). The absorbance of the cells incubated in medium alone was used as a control (survival rate: 100%).

### Cell cycle analysis

The cell cycle distribution of CBD-treated cells was determined by flow cytometry. Fluorescence detection of propidium iodide (PI)-DNA complexes was determined by flow cytometry (FACSCalibur™ flow cytometer BD Biosciences, Heidelberg, Germany). The distribution of cells in different stages of the cell cycle was analyzed using ModFit LT™ software (Scripps Research, La Jolla, CA, USA). A minimum of 30,000 cells was analyzed.

### Apoptosis assay

An Annexin V-FITC/PI double staining assay was performed according to the manufacturer’s instructions (TACS® Annexin V Kits, R&D Systems). Briefly, after 24 h of CBD treatment, cells were collected and incubated with TACS Annexin V-FITC in binding buffer containing propidium iodide (Incubation Reagent) for 15 min at room temperature. Fluorescence intensity was measured using a FACSCalibur™ flow cytometer (BD Biosciences), and the apoptotic rates of CBD-treated cells were analyzed using BD FACSDiva™ software (version 6.1.3; BD Biosciences). A minimum of 10,000 cells were analyzed.

### Mitochondrial membrane potential assay

Mitochondrial membrane potential was determined using the JC-I Mitochondrial Membrane Potential Assay Kit (ab113850, Abcam), according to the manufacturer’s instructions. Fluorescence intensity of cells was measured using a FACSCalibur™ flow cytometer (BD Biosciences). A minimum of 30,000 cells were analyzed.

### Total reactive oxygen species (ROS) measurement

Total intracellular ROS levels were determined using DCFDA/H2DCFDA Cellular ROS Assay Kit (ab133851; Abcam). The DCFDA assay protocol is based on the diffusion of DCFDA/H2DCFDA/DCFH-DA/DCFH into the cell. It is then deacetylated by cellular esterases to a non-fluorescent compound, which is later oxidized by ROS into highly fluorescent 2’,7’-dichlorofluorescein (DCF). Cellular fluorescence (excitation/emission, ~485 nm/~535 nm) was quantified by flow cytometry. A minimum of 10,000 cells were analyzed.

### Caspase activity assay

Measurements of activated caspase-8/9/3 were performed according to the manufacturer’s instructions using FITC-Caspase-3 antibody (345815; R&D Systems) or FITC-Caspase 8/PE-Caspase-9 antibodies (ab65615/ab65618; Abcam). Fluorescence intensity was determined by flow cytometry. A minimum of 10,000 cells were analyzed.

### DNA double strand break labeling by flow cytometry (TUNEL assay)

The TUNEL assay was performed using BrdUTP analysis following the instructions of the assay kit (TUNEL Assay Kit, Thermo Fisher). Fluorescence intensity was determined by flow cytometry (FACSCalibur™ flow cytometer BD Biosciences, Heidelberg, Germany). A minimum of 10,000 cells were analyzed per sample.

### Immunofluorescence

Cells were fixed with 4% (w/v) formaldehyde in phosphate buffered saline (PBS), and then permeabilized by incubation with 0.15% (v/v) Triton X-100 in PBS, after which cells were incubated overnight at 4 °C with monoclonal antibodies to p53 (murine IgG2a clone DO-1, sc-126, Santa Cruz), LC3B—autophagosome marker (rabbit polyclonal, ab48394, Abcam) or Nuclear Factor-Like 2 (Nrf2, murine IgG1 clone A-10, sc-365949, Santa Cruz). Primary antibody binding was detected using IgG (H + L) goat anti-mouse Alexa Fluor™ 488 Superclonal™ recombinant polyclonal secondary antibody (A28175, Invitrogen). Nuclei of labeled cells were counterstained with 40, 6-diamidino-2-phenyIindole (DAPI; 1 µg/ml for 1 min). Fluorescence images were taken using a Leica TCS SP8 confocal microscope. Fiji software (https://imagej.net/software/fiji/, accessed on 22 April 2021) [64] was used for quantification.

### Immunoblot analysis

Cell lysates were prepared, separated by SDS-PAGE and blotting performed as described previously [65]. The protein content was determined using the Pierce™ BCA Protein Assay Kit (Thermo Fisher Scientific). The following antibodies directed against the indicated antigens were used in immunoblotting experiments: p53 (murine IgG2a monoclonal clone DO-1, Santa Cruz), LC3B—autophagosome marker (rabbit polyclonal, ab48394, Abcam), SQSTM1/p62 (recombinant rabbit monoclonal, ab211324, Abcam), p21 Waf1/Cip1 (rabbit monoclonal, 2947, Cell Signaling Technology), CDK2 (recombinant rabbit monoclonal, ab32147, Abcam), PARP1 (rabbit polyclonal, 9542, Cell Signaling Technology), Hsp70 (murine IgG1 monoclonal clone cmHsp70.1, multimmune GmbH), Nrf2 inhibitor keap1 (keap1, rabbit monoclonal, 8047, Cell Signaling Technology), β-Actin (murine IgG2a monoclonal antibody clone AC-74, A2228, Sigma-Aldrich). Primary antibody binding was detected using horseradish peroxidase (HRP)-conjugated rabbit anti-mouse immunoglobulins (P0260, Dako-Agilent) and HRP-conjugated swine anti-rabbit immunoglobulins (P0217, Dako-Agilent) secondary antibodies and a Pierce™ ECL Western Kit (Thermo Fisher Scientific). Blots were digitally imaged using a ChemiDoc™ Touch Imaging System (Bio-Rad). Fiji software (https://imagej.net/software/fiji/, accessed on 22 April 2021) [64] was used for quantifying Western blot signals.

### In vivo tumor xenograft model

Female SCID mice purchased from the Pasteur Institute, Iran was kept under standard laboratory conditions and all experiments were performed according to the requirements of a project license (EE/1401.2.24.105658/SCU.AC.IR) issued by the Faculty of Veterinary Medicine Animal Ethics Committee of the Shahid Chamran University of Ahvaz, Iran. Specifically, animals had ad libitum access to food and water during maintenance under standard conditions (22 °C, 50% relative humidity, and 12 h light/dark cycles). Mice were adapted to the standard housing conditions for 1 week before the start of the experiments. All animal procedures were performed in compliance with the revised Animals Directive 2010/63/EU of the European Union.

Mice were randomly divided into two groups. HCT116 p53wt (2 × 10^6^) and the slower growing HCT116 p53^−/−^ cells (2.8 × 10^6^) were subcutaneously injected into 8-week-old female SCID mice in 100 µl McCoy’s 5 A medium 24 h after a 3 Gy whole-body irradiation. Tumor size, as measured using a caliper and body weight were measured twice per week. CBD (PhytoLab GmbH&Co.KG) was dissolved in a solution (5% DMSO, 5% Tween 80 (P4780, Sigma), 90% stroke-physiological saline solution) injected intraperitoneally (20 mg/kg, i.p.) 5 times a week for 5 weeks in total, from day 5 onwards. Mice were sacrificed on day 40 by isoflurane. Four to six mice were included in each group.

### Immunohistochemistry

Tissue was fixed in formalin overnight and embedded in paraffin. Blocks were sectioned in 2 μm slices and stained with hematoxylin (Mayer’s hematoxylin) and eosin (eosin y-solution 0.5% (v/v) aqueous) to visualize tissue structure according to standard protocols. Caspase-3 staining using the antibody rabbit anti-caspase 3, cleaved (9661, Cell Signaling Technology) was performed to determine the extent of apoptosis. Biotin-conjugated secondary antibodies were incubated for 1 h at room temperature. Nuclear counterstaining was done using hematoxylin.

### Statistical analysis

Data from the in vitro experiments are presented from triplicate independent experiments. Statistical analyses were performed using GraphPad Prism (version 8.0, Graphpad Software, USA). Groups of two were analyzed with Student’s *t* test, groups greater than two with a single variable were compared using one-way ANOVA analysis. Groups greater than two with two independent variables were compared using two-way ANOVA analysis. The value of *p* < 0.05 were considered statistically significant. Data are presented as mean values with standard deviations (SD) or standard error of mean (SEM).

### Supplementary information

Original Data File

Supplementary Figure 1

Supplementary Figure 2

Supplementary Figure 3

## Data Availability

All data are available in the main text or the Supplementary Materials.
