# Supplementary figures and images for: Cannabidiol-induced crosstalk of apoptosis and macroautophagy in colorectal cancer cells involves p53 and Hsp70
Source: Cell Death Discov. 2023 Aug 5;9:286. doi: 10.1038/s41420-023-01578-9 (PMC10403543; doi:10.1038/s41420-023-01578-9)

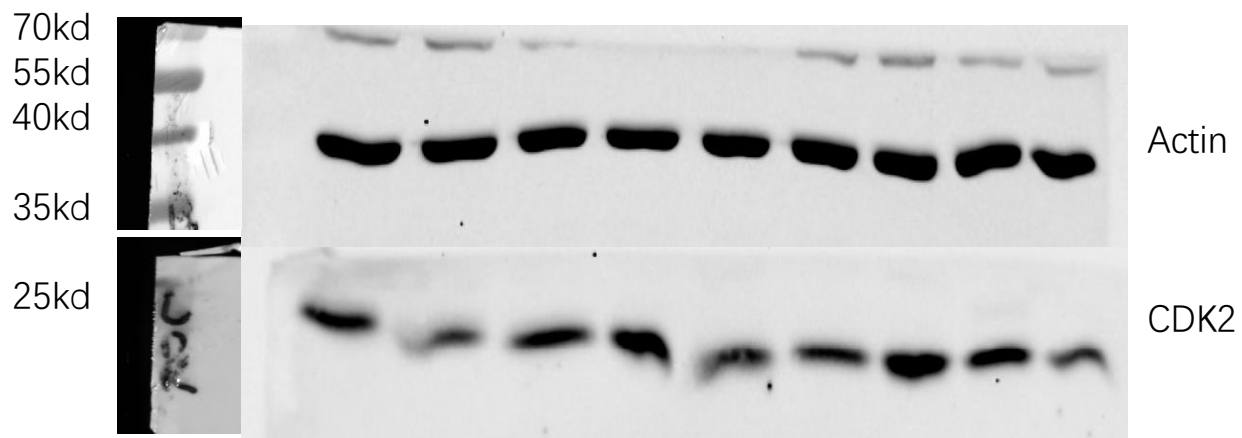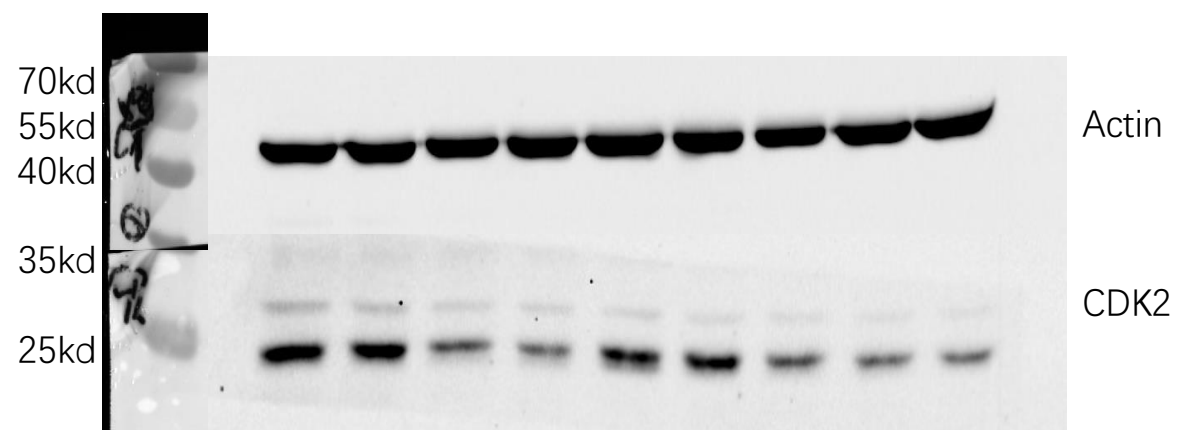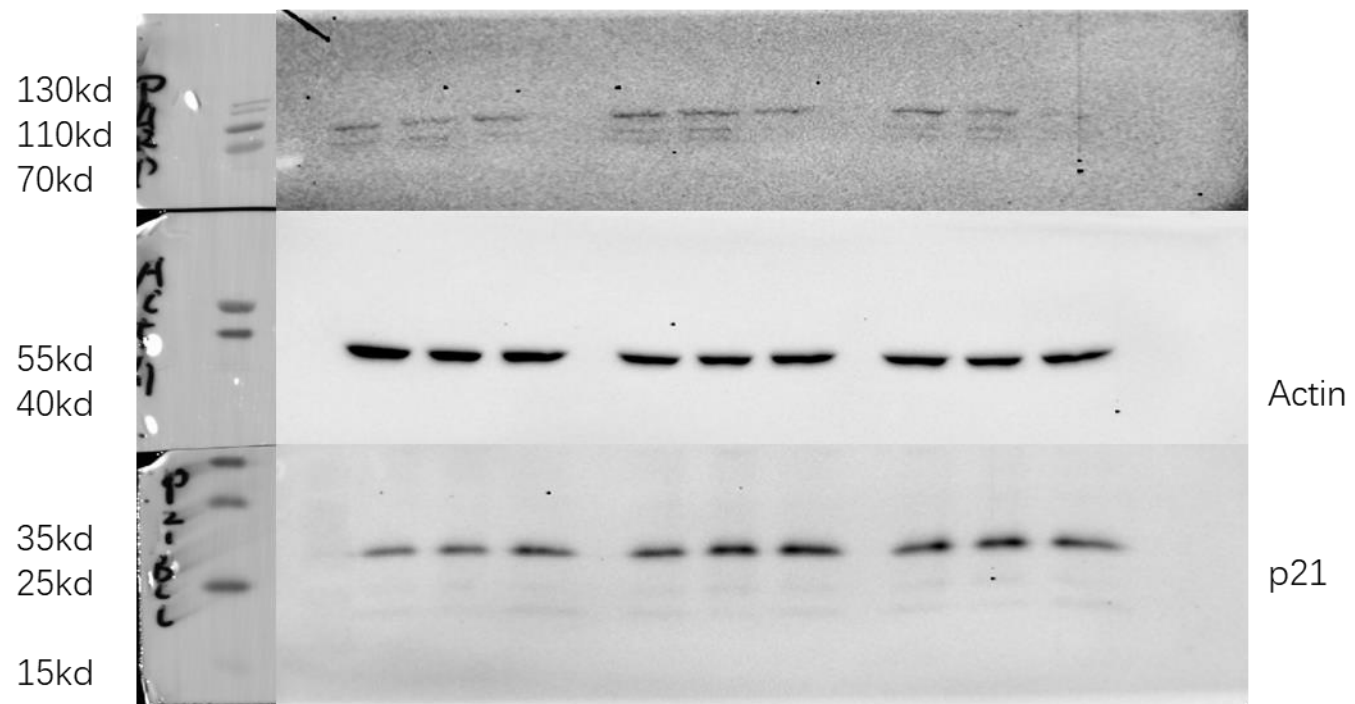

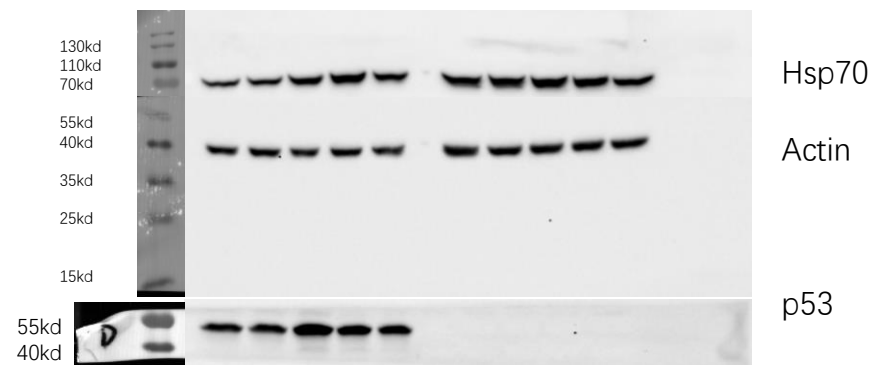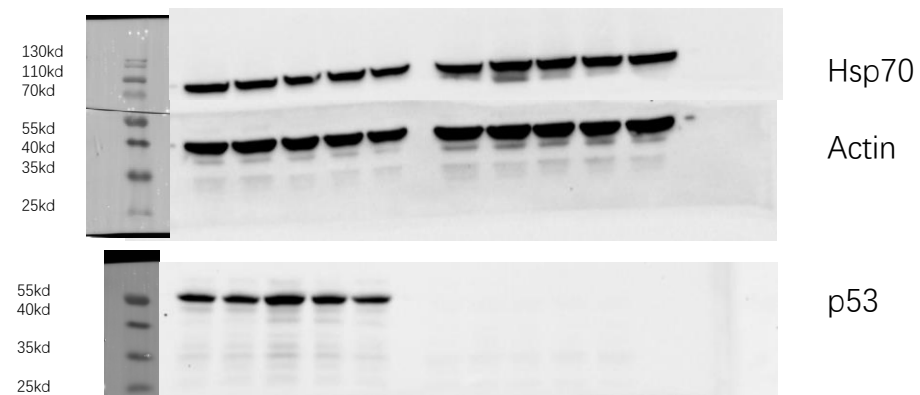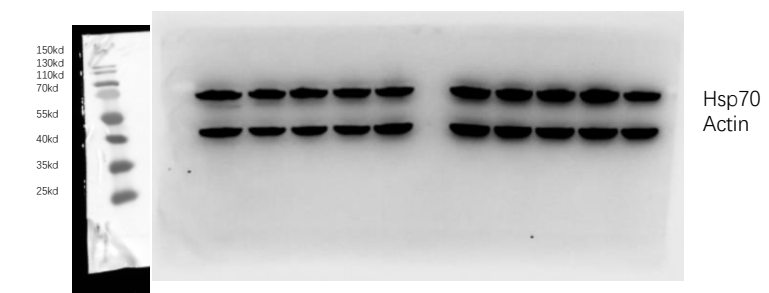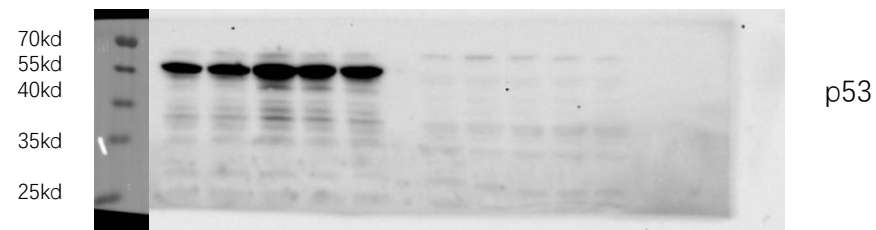

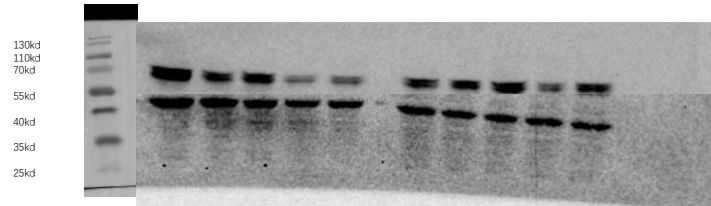

Keap1  
Actin

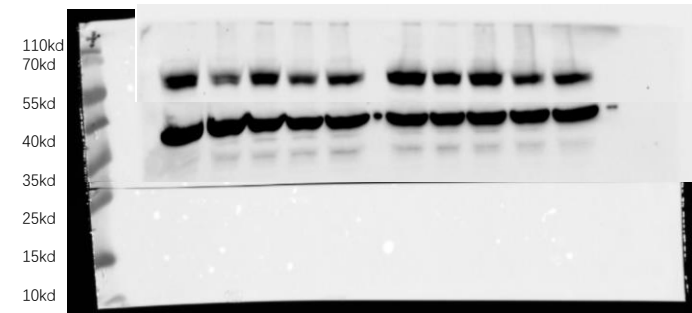

Keap1  
Actin

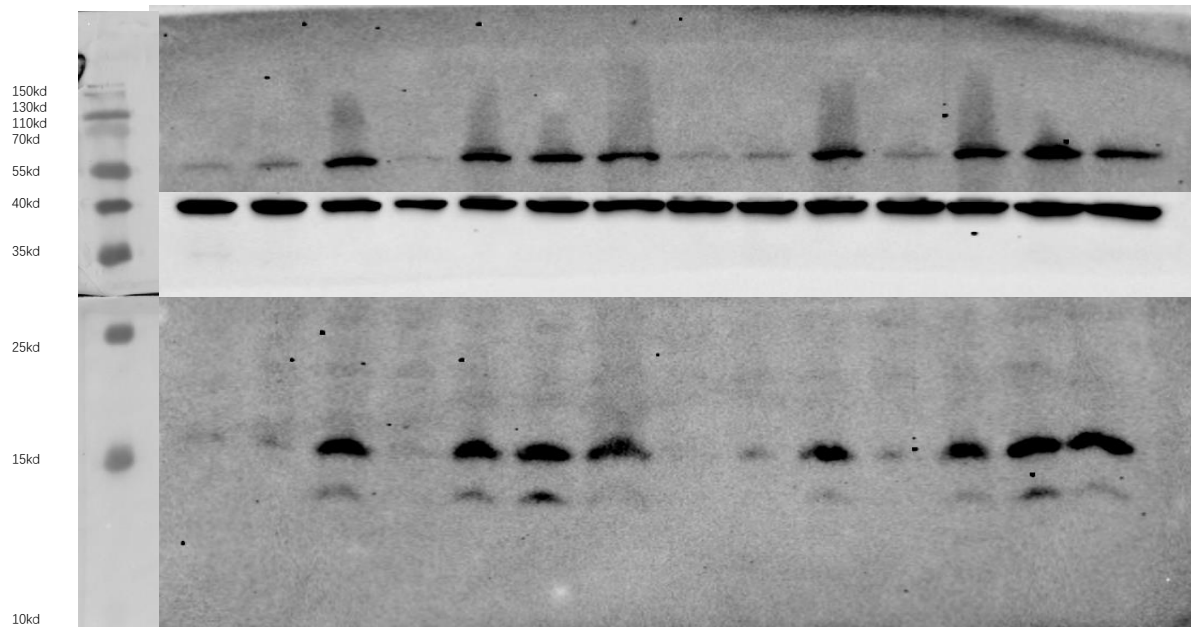

P62  
Actin  
LC3B

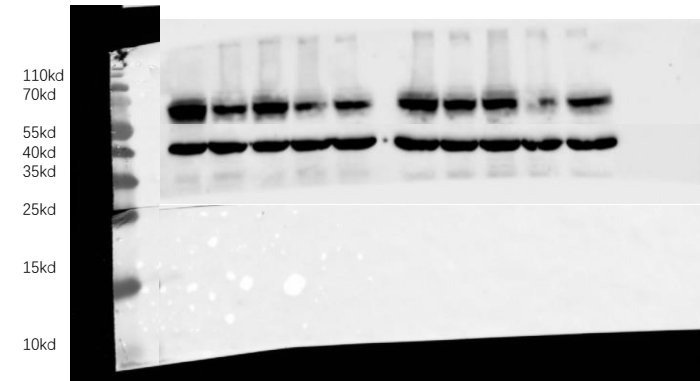

Keap1  
Actin

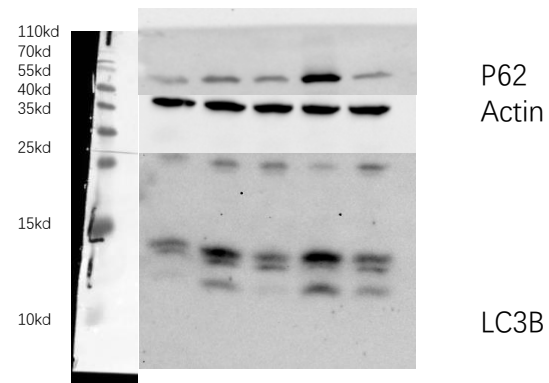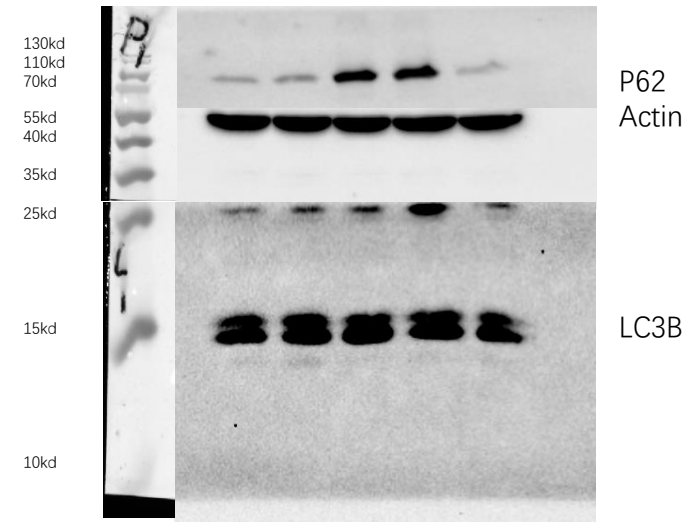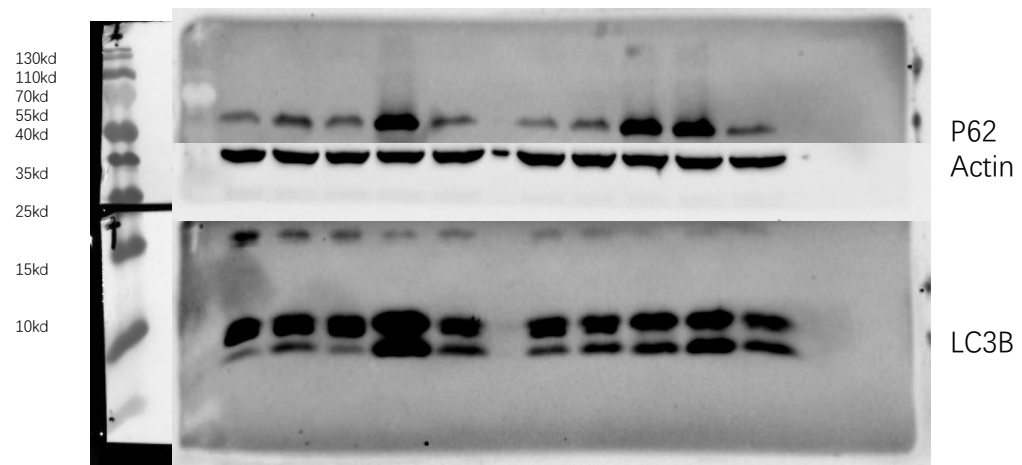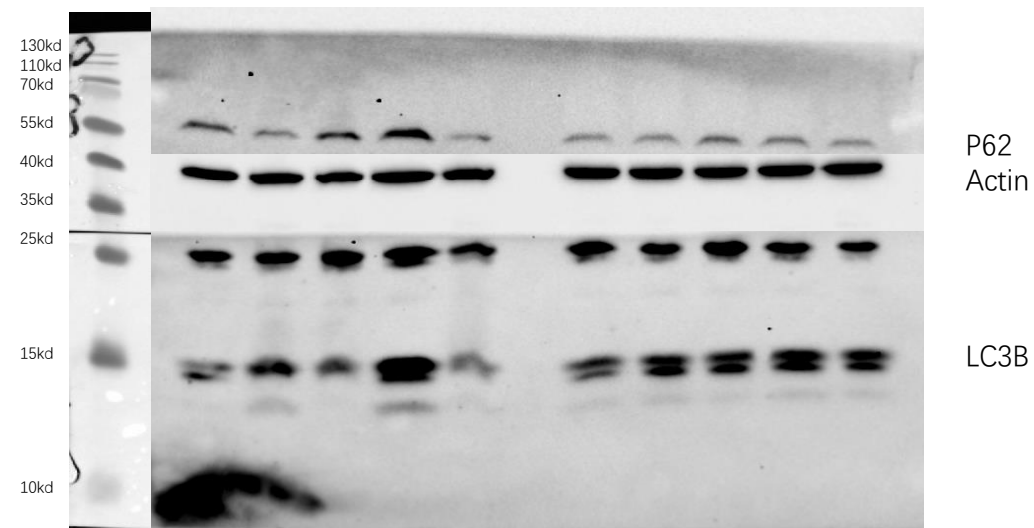

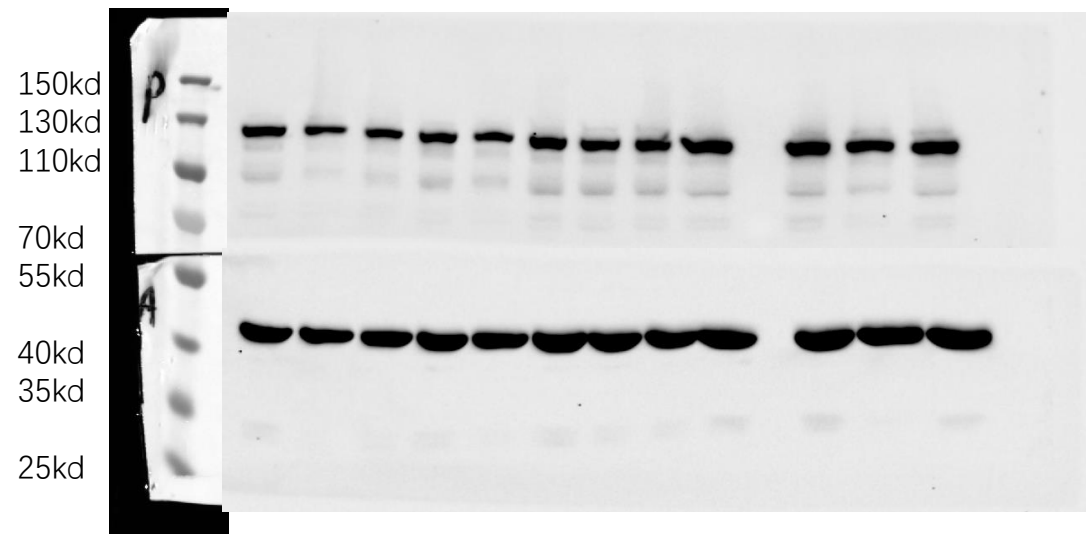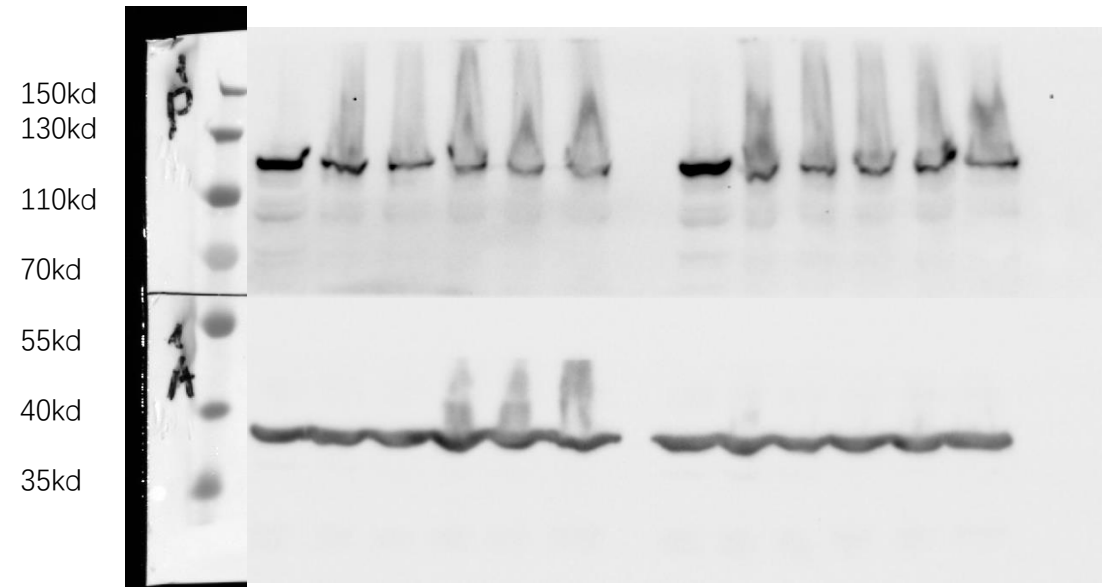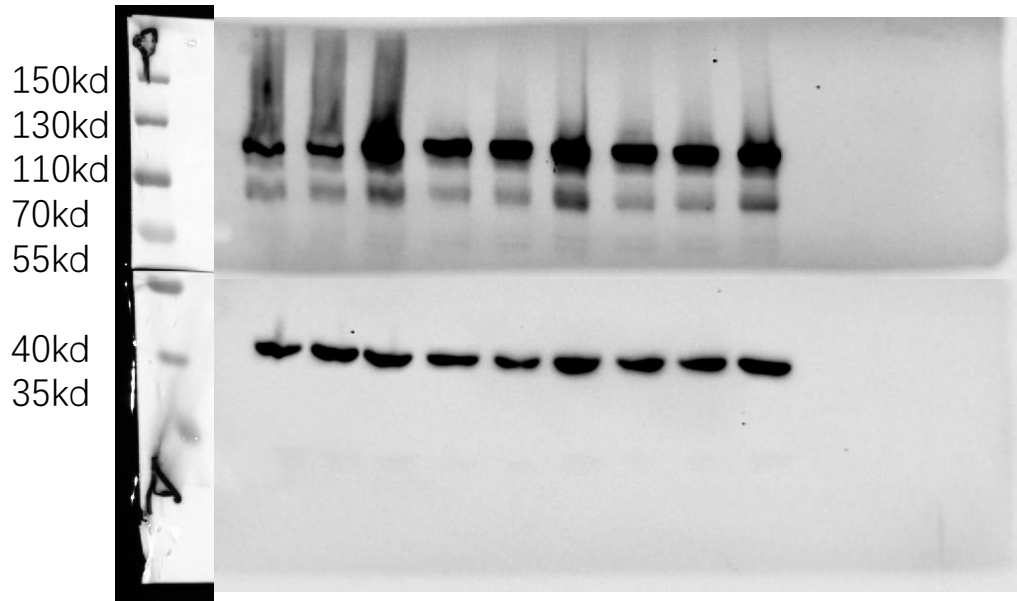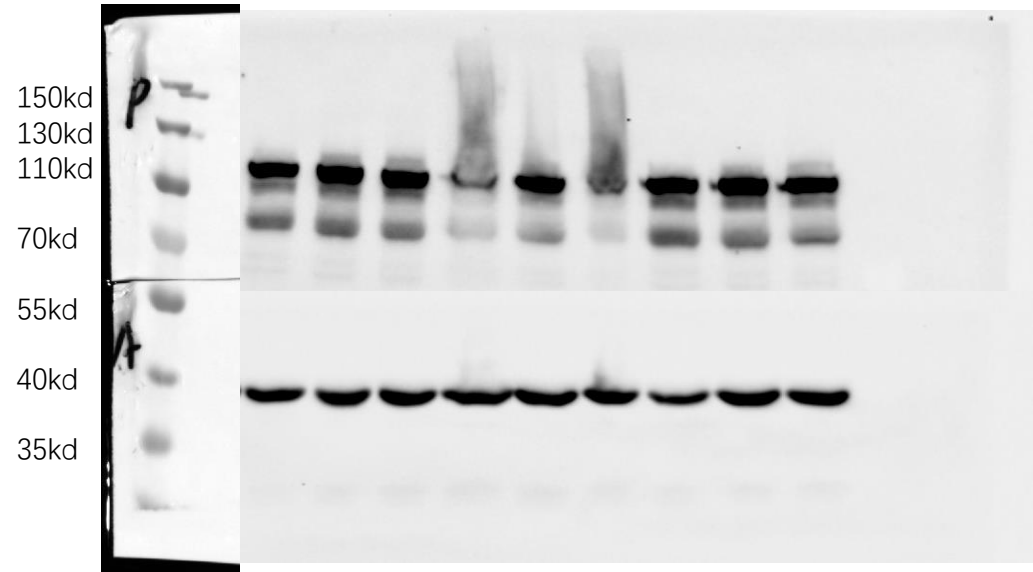

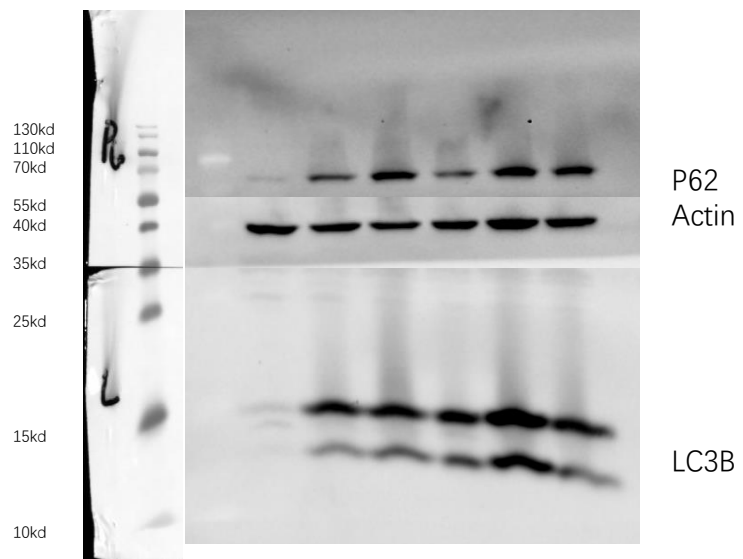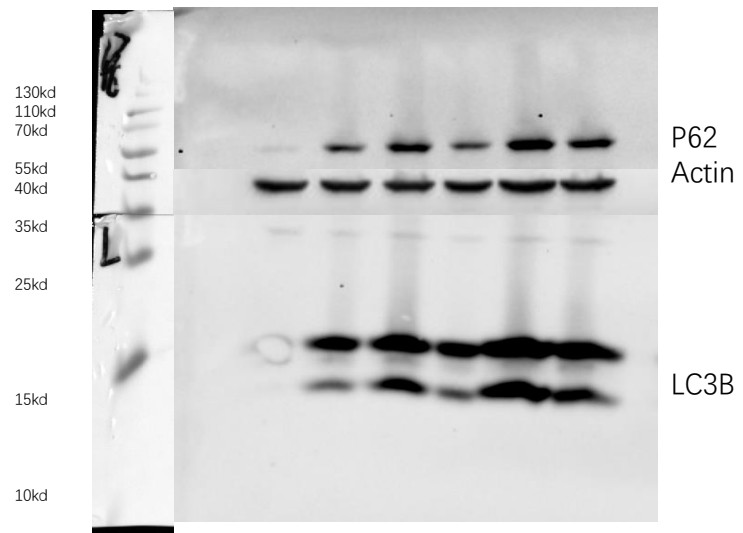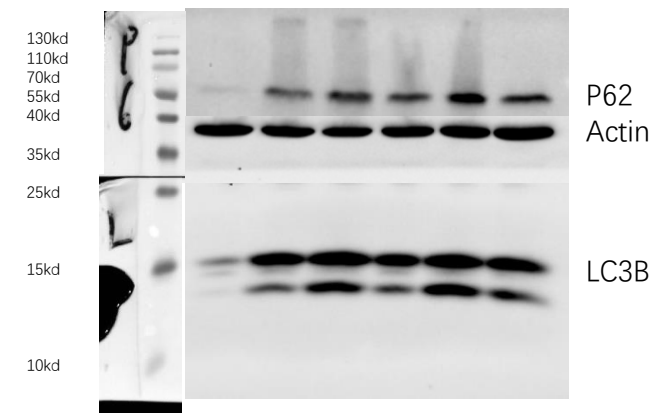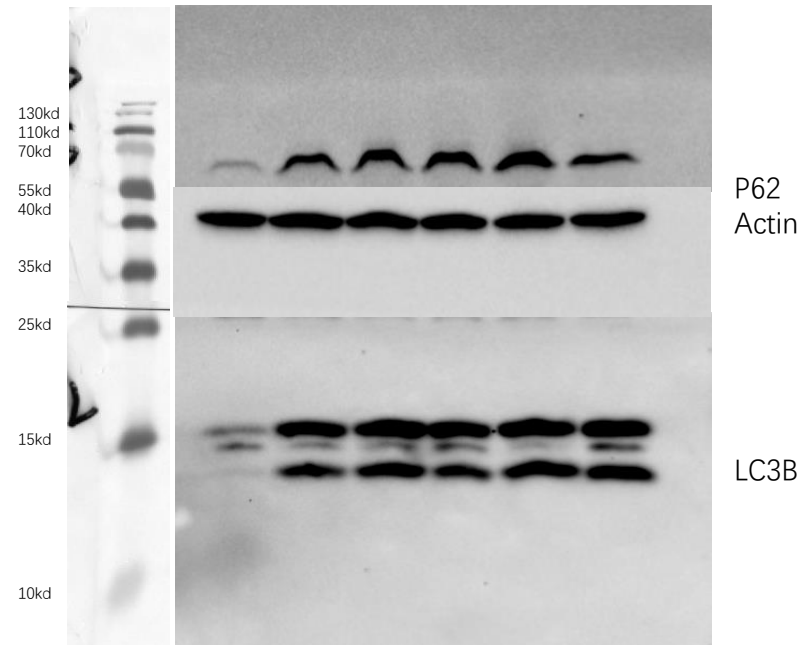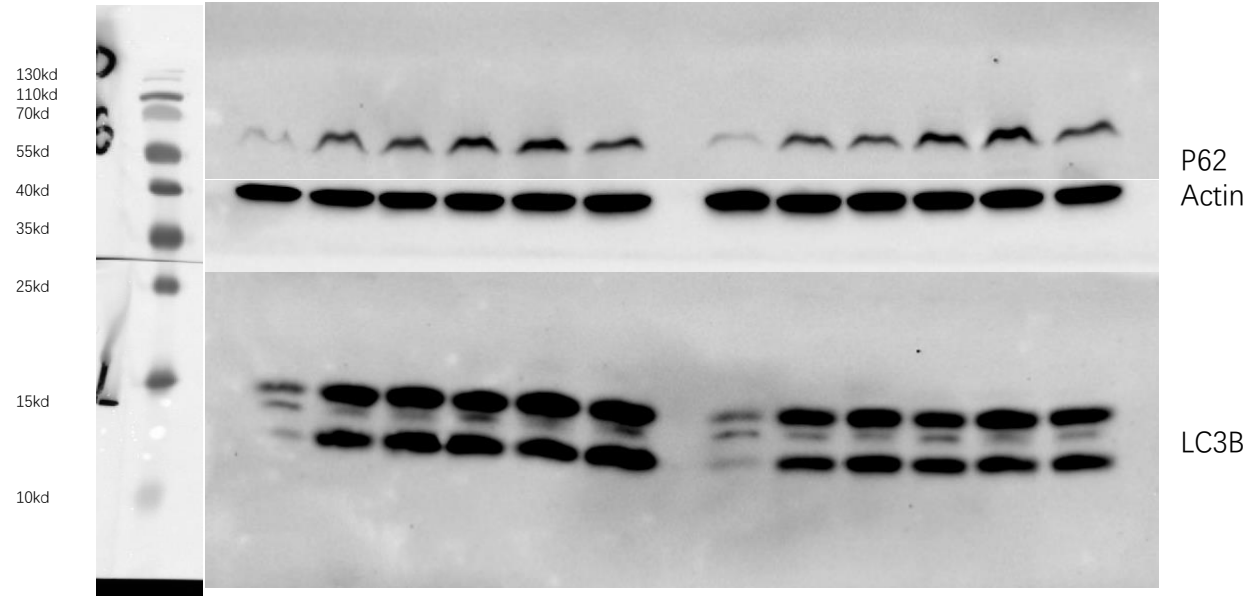

Supplement: Supplementary file 1 — Original Data File [file 41420_2023_1578_MOESM1_ESM.pdf]

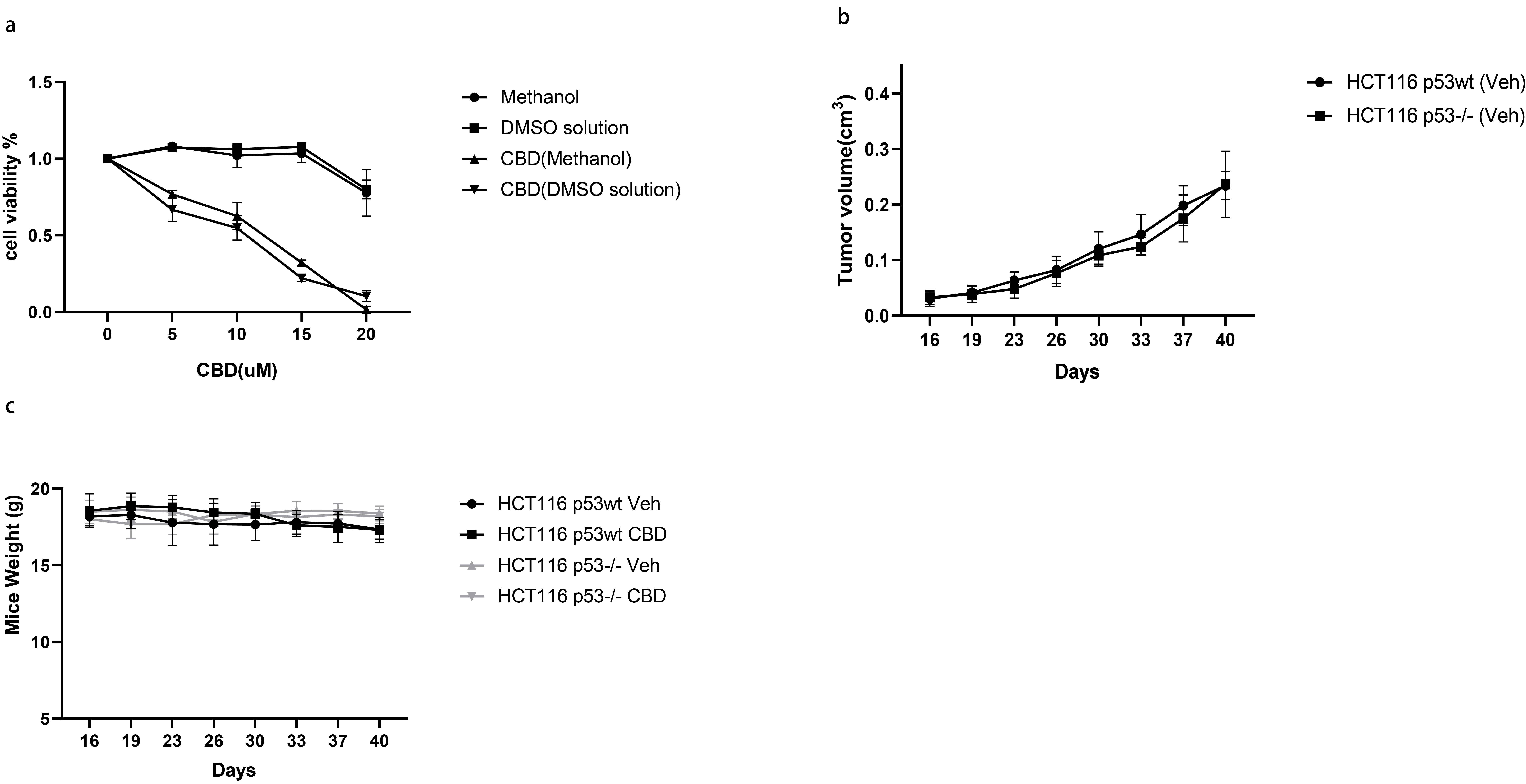

Supplement: Supplementary file 2 — Supplementary Figure 1 [file 41420_2023_1578_MOESM2_ESM.tif]

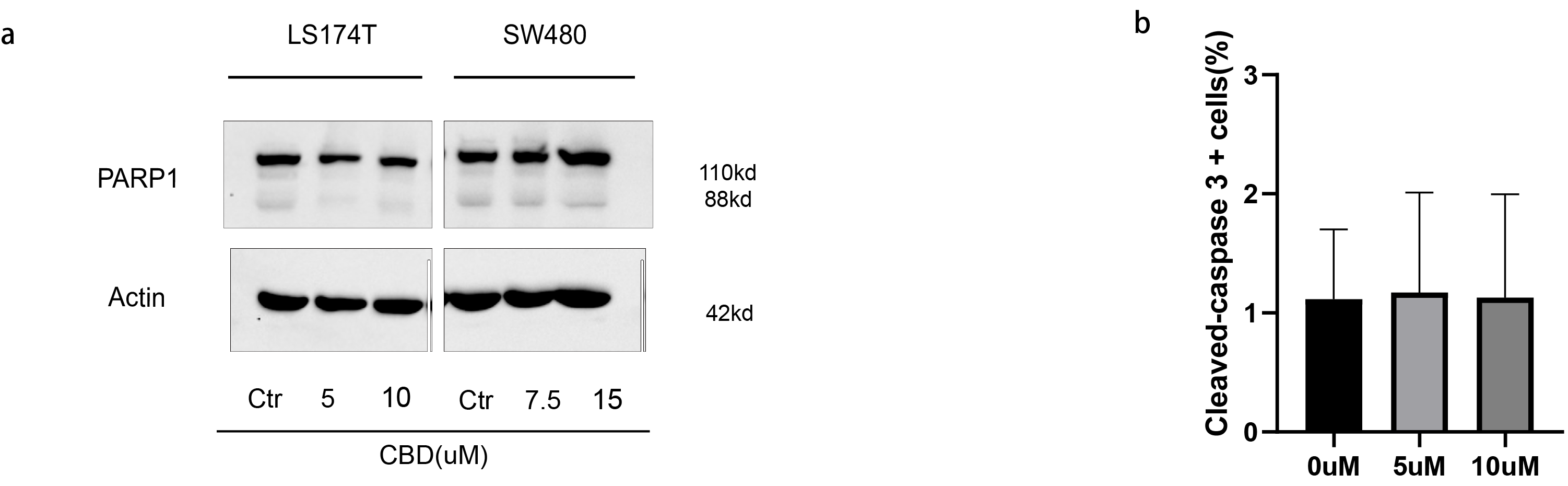

Supplement: Supplementary file 3 — Supplementary Figure 2 [file 41420_2023_1578_MOESM3_ESM.tif]

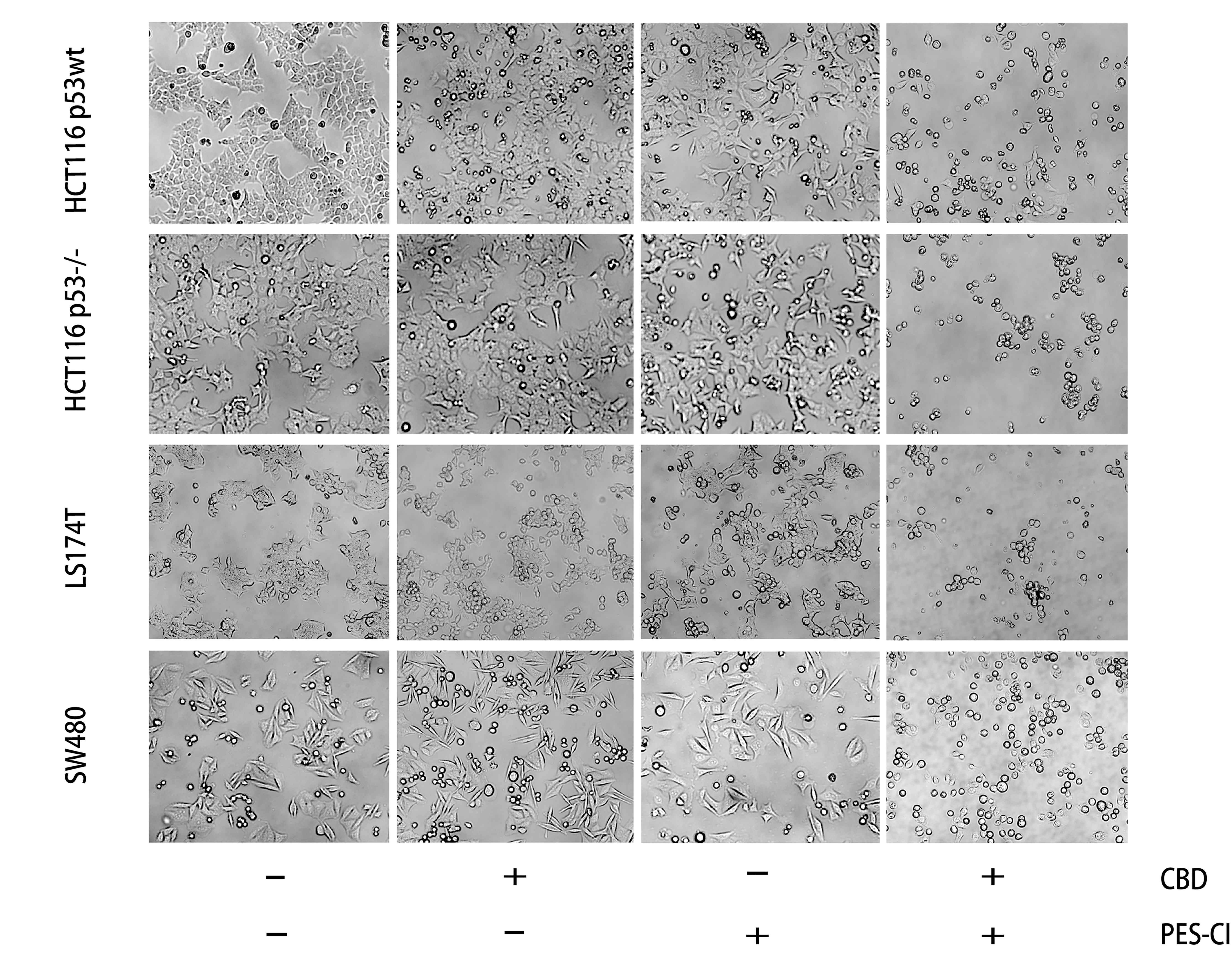

Supplement: Supplementary file 4 — Supplementary Figure 3 [file 41420_2023_1578_MOESM4_ESM.tif]
